# A systematic review of engagement reporting in remote measurement studies for health symptom tracking

**DOI:** 10.1038/s41746-022-00624-7

**Published:** 2022-06-29

**Authors:** Katie M. White, Charlotte Williamson, Nicol Bergou, Carolin Oetzmann, Valeria de Angel, Faith Matcham, Claire Henderson, Matthew Hotopf

**Affiliations:** 1 Department of Psychological Medicine, King’s College London, London, UK; 2 King’s Centre for Military Health Research, King’s College London, London, UK; 3 Department of Psychosis Studies, King’s College London, London, UK; 4 Health Service & Population Research Department, King’s College London, London, UK; 5 South London and Maudsley National Health Service Foundation Trust, London, UK

**Keywords:** Preventive medicine, Predictive markers, Epidemiology, Psychology

## Abstract

Remote Measurement Technologies (RMTs) could revolutionise management of chronic health conditions by providing real-time symptom tracking. However, the promise of RMTs relies on user engagement, which at present is variably reported in the field. This review aimed to synthesise the RMT literature to identify how and to what extent engagement is defined, measured, and reported, and to present recommendations for the standardisation of future work. Seven databases (Embase, MEDLINE and PsycINFO (via Ovid), PubMed, IEEE Xplore, Web of Science, and Cochrane Central Register of Controlled Trials) were searched in July 2020 for papers using RMT apps for symptom monitoring in adults with a health condition, prompting users to track at least three times during the study period. Data were synthesised using critical interpretive synthesis. A total of 76 papers met the inclusion criteria. Sixty five percent of papers did not include a definition of engagement. Thirty five percent included both a definition and measurement of engagement. Four synthetic constructs were developed for measuring engagement: (i) engagement with the research protocol, (ii) objective RMT engagement, (iii) subjective RMT engagement, and (iv) interactions between objective and subjective RMT engagement. The field is currently impeded by incoherent measures and a lack of consideration for engagement definitions. A process for implementing the reporting of engagement in study design is presented, alongside a framework for definition and measurement options available. Future work should consider engagement with RMTs as distinct from the wider eHealth literature, and measure objective versus subjective RMT engagement.

Registration: This review has been registered on PROSPERO [CRD42020192652].

## Introduction

Remote Measurement Technologies (RMTs) provide real-time, longitudinal health tracking by collecting frequent questionnaire, physiological and behavioural data outside and between traditional clinic or research assessments^1^. RMTs form a subsection of the electronic health (eHealth) movement, which utilises mobile or online technologies to improve patient outcomes^2^. Active RMT (aRMT) measures comprise smartphone applications (apps) for symptom reporting and can be combined with mobile or wearable sensors for passive RMT (pRMT) data collection^3–5^. RMTs hold great potential for the management of chronic health conditions. First, they can provide unbiased data on symptom fluctuations and clinical state. Remote symptom assessments have been validated against in-person measures across physical and mental health conditions^6–8^. By measuring symptoms remotely, it is also possible to gain more detailed longitudinal information than is possible through less frequent clinic or research assessments. Second, multi-parametric RMT data may provide the temporal resolution needed to detect indicators of future relapse or remission in long-term conditions^9^. Preliminary work has suggested that remote monitoring of depression and anxiety may prove valuable in predicting future symptoms^10^. The use of RMTs could potentially revolutionise research, clinical practice, and self-management in chronic diseases^11^.

The promise of RMTs depends almost entirely on user engagement. Engagement can be broadly understood as a multi-stage construct indicating the extent to which a resource is actively used^12^. In research, low initial engagement in RMT studies, i.e., uptake, increases the risk of selection bias, lowers statistical power, and reduces the external generalisability of results^13^. Sustained engagement, i.e., retention and ongoing adherence to research protocols, is essential to ensure that the resulting datasets in research studies are complete and therefore allow identification of patterns of symptom change^14^. Describing and understanding the drivers of participants’ engagement with RMTs in research is necessary to determine the success of future real-world implementation of RMTs in clinical services.

The current state of engagement with RMTs is unclear. Qualitative research suggests acceptance of the use of technology for symptom monitoring in conditions such as depression^15^, multiple sclerosis^16^, epilepsy^17^, arthritis^18^ and fibromyalgia^19^. In practice however, reported engagement statistics are hugely heterogeneous. Dropout rates for studies of remote monitoring of various lengths in depression have been estimated at 3.6%^20^ and 26.2%^21^ in separate reviews. A large caveat to synthesising findings is the current lack of standardisation in the measurement and reporting of participant engagement. Simblett et al.^22^ found that a variation of idiosyncratic, non-comparable engagement measures was reported across 33 studies, which included dropout, adherence rates, and usage statistics. This heterogeneity was noted to have ‘severely limited quantified conclusions’. Two recent systematic reviews on using RMT data to monitor symptom change cited methodological differences and inadequate missing data reporting as key barriers to performing meta-analyses^23,24^. There is a clear need to understand how engagement is reported in the current RMT literature, in order to promote reproducibility of results and ensure that future meta-analyses can compare like with like.

A body of work in the wider eHealth field has made significant progress in the standardisation of engagement reporting. The publication of guidelines such as CONSORT-EHEALTH^25^ and STROBE^26^ has considerably improved reproducibility, with regards to the reporting of recruitment, adherence and attrition. Systematic reviews of eHealth studies have further explored how authors report on engagement. Sieverink et al.^27^ recommended that the inclusion of a ‘definition of intended engagement, justification with theory or rationale, and corresponding measurement’ was crucial for comparing engagement across technologies^27^. Perski et al.^28^ conducted a systematic review using critical interpretive synthesis to further explore how engagement with digital behaviour change interventions (DBCIs) had been defined and measured. They proposed an integrative definition, comprising *objective* (the extent of usage, e.g., adherence) and *subjective* (experiential factors, e.g., attention, interest) engagement concepts. These subjective and objective components were underpinned by corresponding measurements. This framework has since been used to synthesise the reporting of engagement in eHealth interventions for depression^29^. Thus, an exploration of varying definitions and measurements of engagement has been key to standardising the findings of the wider eHealth literature. It is currently unclear how far these findings apply to RMT work specifically.

This systematic review aims to explore the current state of engagement reporting in studies using RMTs to track symptoms in physical and mental health conditions. Our broad aim is to describe the extent to which engagement is defined, measured, and reported in the literature. There are three main objectives:

1. To present a quantitative synthesis of the proportion of studies which report on any form of engagement definition or measurement.
2. To synthesise studies reporting on engagement with regards to the following questions:
  - How has engagement been defined in the selected literature?
  - How has engagement been measured in the selected literature?
3. To present recommendations for the standardisation of future work in this field

Our findings will aid in understanding the current trends in the field, and promote standardisation for future work, syntheses, and evaluation of RMT research.

## Results

### Summary of search results

**Fig. 1:**
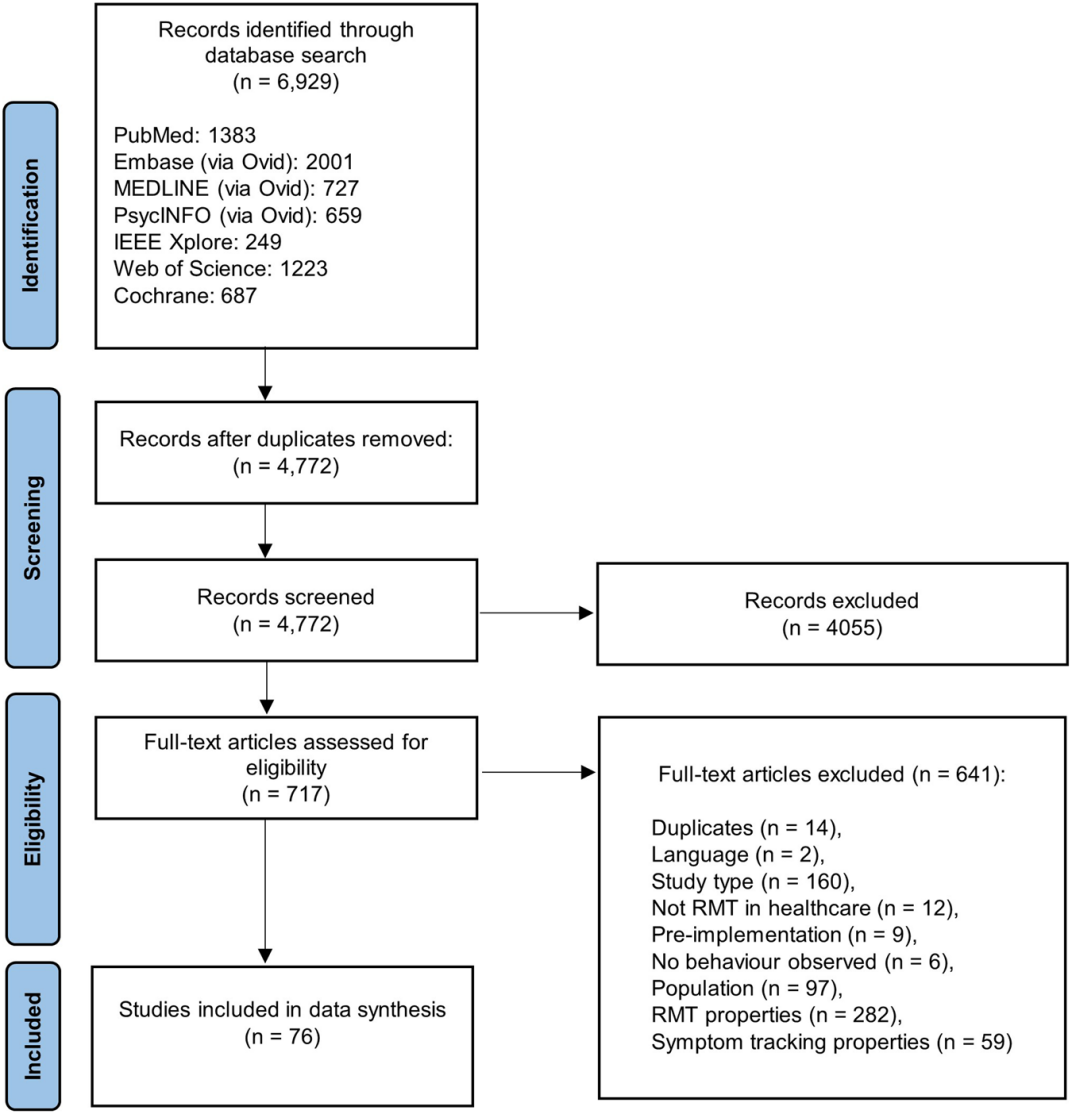
PRISMA flow diagram of included studies. Seven databases were searched to ensure relevant fields were covered. The flow diagram lists reasons for exclusion of articles from the final sample of *n* = 76.

The electronic database search yielded 6929 articles, reduced to 4772 after removing duplicates across databases. Of the full texts screened, 76 met the inclusion criteria and were included in the data synthesis (Fig. 1). Sixty studies were described across the 76 papers.

Characteristics of the included papers are described in Supplementary Table 1. The most common conditions of study were bipolar disorder (*n* = 23), depression and/or anxiety disorder (*n* = 11), chronic pain (*n* = 8), cancer (*n* = 5), and psychosis (*n* = 4). Forty one papers (54%) reported on RMT use in a research context, 22 (29%) on RMTs for self-management and 13 (17%) on the use of RMTs for clinical support. aRMT only was used in 45 (59%) papers, where 31 (45%) papers used a combination of aRMT and pRMT methods. Symptoms were tracked for a median of 56 days (range = 794 days). Common symptoms tracked included mood, pain, sleep, fatigue, exercise, and daily activity levels.

### To what extent has engagement been reported in the literature?

Of the 76 papers included, a large majority (*n* = 74, 97%) reported on engagement in some form. The two papers that did not report on engagement^30,31^ are secondary analyses and cite previous feasibility papers. Of the 74 papers that did report on engagement, 48 (65%) did not include a definition of engagement. Twenty-six (35%) papers included both a definition and corresponding measurement of engagement. All but one of the papers that included a definition and measurement noted engagement as a main aim of the paper; Greer et al.^32^ aimed to test the effect of a smartphone app, of which symptom monitoring was one component, on adherence to cancer treatment. Of the 30 papers (39.5%) that noted engagement as a main aim, 5 (17%) did not include a definition of engagement.

### How has engagement been defined and measured in the literature?

We identified five synthetic constructs for engagement definition: (i) objective definition, e.g., feasibility, (ii) subjective definition, e.g., usability, (iii) utility, (iv) app usage, and (v) engagement with the mobile research protocol. Four synthetic constructs were developed for engagement measurement: (i) engagement with the research protocol, (ii) objective RMT engagement, (iii) subjective RMT engagement, and (iv) interactions between objective and subjective RMT engagement. Table 1 describes the number of papers that included each of the definition and measurement constructs.

**Table 1:** *N* (%) of papers that included each of the engagement definition and measurement constructs.

Characteristic	*N*	%
Engagement as a main aim^a^	30	39.5
Gave a definition of engagement^b^	26	35.1
Objective, e.g., feasibility^b^	18	24.3
Subjective, e.g., usability^b^	11	14.9
Utility^b^	3	4.1
App usage^b^	4	5.4
Research protocol	1	1.4
Reported a measure of engagement^a^	74	97.4
With the research protocol^b^	65	87.8
Objective RMTs^b^	64	86.5
Subjective RMTs^b^	27	36.5
Combined objective and subjective RMTs^b^	22	29.8

^a^Denominator of 76. ^b^Denominator of 74. Categories are not mutually exclusive.

Papers used a range of, often interchangeable, definitions for engagement. The most cited term was ‘feasibility’. For many studies, feasibility was conceptualised as objective compliance with, or adherence to, symptom monitoring^33–37^, such that higher completion rates signalled greater feasibility. Depending on the context of RMT use, feasibility either represented (i) the ability of users to use an app to complete the monitoring, or (ii) the ability of RMTs to collect sufficient data for assessment of the cohort in question. Terms such as ‘usability’, ‘acceptability’ and ‘satisfaction’ were often used in addition to feasibility. These tended to be more subjective concepts, often paired with rating scales and questionnaires to understand how far users felt the technologies were appropriate for use. Two studies further understood engagement as ‘perceived utility’ or ‘usefulness’ of the RMT for disease management^38,39^. Other studies defined app usage data as a proxy for engagement. Selter et al.^38^ sought to understand ‘patient engagement’ on the basis of the frequency and quality of interaction (active vs. passive) with components of the self-monitoring system, and^40^ identified patient preference for RMT type by usage data. One paper defined engagement as “participant willingness to engage in mobile research protocols”^41^ (pg. 14). Overall, a large proportion of studies used multiple conceptualisations of, and terms for, engagement in the same paper, often with little justification for why they had been chosen or how they complemented each other.

What follows comprises a narrative synthesis of each synthetic construct for the measurement of engagement across the included papers.

### Engagement with the research protocol

A large majority of papers (88%, *n* = 65) reported on engagement in reference to recruitment, retention and withdrawal from RMT studies. Where generic reasons for declining research participation, such as lack of time and/or lack of interest, were widely reported, some studies also reported on RMT-specific reasons: unwilling to use/switch to trial smartphone^32,42–45^, concerns about remote data collection^42,45,46^, having ‘no need’ to monitor symptoms alongside treatment^47^. One study^48^ calculated the percentage of participants who declined to participate explicitly based on the RMT nature of the research, using data on previous non-response to clinic and research opportunities.

Reasons for participant dropout followed a similar vein. Technological issues, for example an inability to download the study app^49^, phone updates^33^ and software malfunctions with study apps^33,36,37^, were the most reported reasons for non-completion of a study. However, many papers did not differentiate these from non-RMT related reasons for dropout, e.g., death or hospitalisation, or failed to provide reasons at all. Seven studies^2,35,41,49–52^ investigated whether dropout was associated with sociodemographic or clinical variables, and one further explored the association between dropout and daily access to data, as a main aim of the study^50^. Two studies reported on likelihood of dropout over time, finding opposing results^35,53^. Understanding the factors associated with decline and dropout over time provided insights into interest in RMTs for support alongside clinical care^47,48^.

There was little consensus over what constituted researcher-initiated withdrawal from the study. Again, there was a lack of distinction made between RMT and non-RMT related factors. Some studies reported on the percentage of participants completing the full number of days of remote monitoring^34,54,55^, where others focused on completion of a sufficient, often arbitrary, number of daily assessments. Participants were withdrawn or excluded from analyses if they did not submit any daily assessments^33,37,56^ or never used the app^36^. Two papers^51,57^ defined withdrawal based on failure to complete a minimum amount of ESM assessments during the study period: 33% and 30% respectively. A failure to attend appointments for additional, in-person questionnaires was also a reason for withdrawal^36,43,58^. Retention rates were largely discussed in terms of the resulting impact on the final dataset for analyses. Overall, a lack of standardisation in the measurement of engagement with the research protocol, coupled with the merging of technological and non-technological factors, suggests that attempts to provide accurate information on dropout and retention rates in RMT studies would be difficult.

### Objective engagement with RMTs

Alongside engagement with the research protocol, studies also reported engagement with the RMTs themselves. 87% (*n* = 64) of papers conceptualised engagement with RMTs as an objective measure, e.g., direct behaviour whilst using the device. First, studies reported on the total, raw number of assessments sent and responded to, or the number of symptoms tracked, during the study period in the sample as a whole. This was further broken down into ‘per participant’ measures: the mean or median average number of daily aRMT assessments completed across the study period^32,33,49,53,54,59,60^, or the average number of assessments completed within each day^61–63^, depending on the frequency of the aRMT tasks. Some studies also reported the number of days in which participants provided self-monitoring data^37,46,53,54^, though at times it was unclear whether this referenced only the days where data was recorded, or the total number of days during which the participant remained in the study.

Second, symptom tracking compliance rates were reported. There was little standardisation in how these were measured. ‘Compliance rate’ was generally conceptualised as the percentage of aRMT assessments completed over the total available to be completed and was used interchangeably with the terms ‘response rate’, ‘adherence’, and ‘completion’. Many studies simply reported the average percentage compliance, often with accompanying measures of variance, across the sample for aRMT assessments and associated clinical measures. Some instead reported on the percentage of days in which self-monitoring was adhered to^64–66^ (assuming that self-monitoring was assessed daily). Some also measured compliance against set criteria, for example the percentage of participants who completed all assessments^42,67–69^, or percentage completion at longitudinal time points; Jamison et al.^33^ reported the percentage of participants who completed at least 30, 60 and 90 daily assessments respectively. Four studies assessed the variation in compliance across time of day^69–72^. Of the 26 studies which included passive tracking, five reported on some form of pRMT compliance^33,40,56,73,74^. These included the percentage of participants logging daily Fitbit data and the percentage of days in which ‘complete’ actigraphy/activity data was logged. One study defined multi-parametric adherence as the proportion of study days with at least 50% of daily self-report questionnaires completed and 12 h of logged activity tracking, chosen to align with future research and clinical goals^40^. Crucially however, most studies gave little or no justification for their measurement of compliance, or the conclusions that were drawn from it.

Third, app usage statistics were used when discussing engagement. For studies which required participants to remotely enrol, percentage of successful study app downloads was a fundamental indicator of engagement^33,49,50^. Other reported usage statistics throughout the study period included total app use (per day, per week or per the study period)^32,42,52,71,75,76^ and number of times the app was launched by the user, be that self-initiated^75^ or in response to prompts^36,60^. One multi-parametric study directly compared the number of days spent engaging with the app with that of the Fitbit^40^. Studies which included RMT symptom tracking as a component of a behaviour change app also reported on in-app module viewing^38,47,77,78^. The impact of app usage was considered by three studies: (i) minutes and days of app use accounted for a large percentage of variance in an ‘app engagement factor’^32^, (ii) viewing in-app symptom visualisations correlated with aRMT and pRMT adherence^40^, and (iii) longitudinal app use was considered to reflect ‘satisfaction and interest’^76^.

### Subjective engagement with RMTs

An exploration of subjective engagement, e.g., indirect user experience during or after interacting with the device, was included as an adjunct to objective engagement in 27 (37%) papers. Many studies used quantitative measures, usually in the form of Likert scales, to measure participant experiences of engaging with remote self-monitoring. These were administered at study end, apart from one study^72^ which issued a usability scale after week one in order to assess early technological problems. Four studies also assessed corresponding clinicians^33,44,68,79^. Scales included a wide range of questions surrounding two main themes: (i) usability of, or satisfaction with, the technology itself, and (ii) utility of the technology for symptom management. Technology usability assessed ease of use, helpfulness of reminders and navigation of the app interface. Utility of the technology focused more on the use of the technology for symptom management and communication with clinical care teams, and intentions for future use. Most studies included questions covering both themes to some extent, however crucially these were generally assessed as a combined ‘satisfaction’ variable (apart from^42^ which used two distinct scales to measure technology obtrusiveness and clinical utility respectively). Studies either combined several, validated scales in one paper, including the System Usability Scale (SUS)^36,69,75,80^ and Technology Assessment Model (TAM)^75,81^, or used author-developed scales, due to a lack of ‘psychometrically evaluated measures of user acceptance’ of RMTs^81^.

Qualitative measures of subjective engagement also explored participants’ experiences. Five studies conducted semi-structured interviews with participants^34,40,42,78,82^, and one with care managers^42^. Other studies reported more vaguely on ‘evaluative feedback’^49,65,83^, ‘response to open-ended questions’^36,39,84^ or ‘in-app free text’^78^ collected throughout follow-up. Qualitative methods were nearly always collected in conjunction with quantitative usability scales, or as a section of the usability scale itself, however one study^80^ recorded questions and difficulties reported by participants to the researchers during the initial demonstration of the technology. Emerging themes covered overviews of experience, challenges and benefits of RMTs for symptom management, and suggestions for improvement.

### Interactions between objective and subjective engagement with RMTs

Most studies reported on objective and subjective engagement either in isolation, or as one, combined ‘engagement’ variable. However, 22 papers (30%) explored the link between the objective and subjective measures used.

Some studies reported on the resulting, subjective effects of using RMTs for symptom monitoring in the study (either as a main aim or an additional outcome). Where the technology took the form of an intervention, quantitative analyses explored associations between RMT use and changes in main outcome variables, including symptom severity^32,33,37,43,49,62,76,85^, physical activity^33,37,49^, receipt of treatment^32,86^, and medication uptake^32^. Other studies reported on the impacts of remote self-monitoring from a more exploratory standpoint. The following qualitative themes were suggested: identification, self-awareness and mindfulness of symptoms and/or emotional health^34,36,40,42^, adapting self-management strategies^42,65^, being seen ‘as a person’^78^, having access to a ‘safety net’ of professional support^51,78^. Only one study suggested a perceived negative effect of RMT use; viewing real-time mood forecasting might lead to a self-fulfilling prophecy, worsening mood^87^.

Five studies conducted correlational analyses on the relationship between objective and subjective variables^33,37,49,53,84^. Participants who reported ‘liking’ an RMT app, or a higher degree of satisfaction, tended to submit more daily assessments^84^ and have a higher duration of compliance (consecutive days with recorded data)^53^. Two studies from the same author found significant Pearson product moment correlations between higher daily assessment count and app satisfaction, across both a 3-month and 6-month time point^33,37^. A consideration of the interaction between both forms of engagement was considered essential for encouraging future use of RMTs for remote medical and psychological assistance^37^.

### An integrative framework for the reporting of engagement

**Fig. 2:**
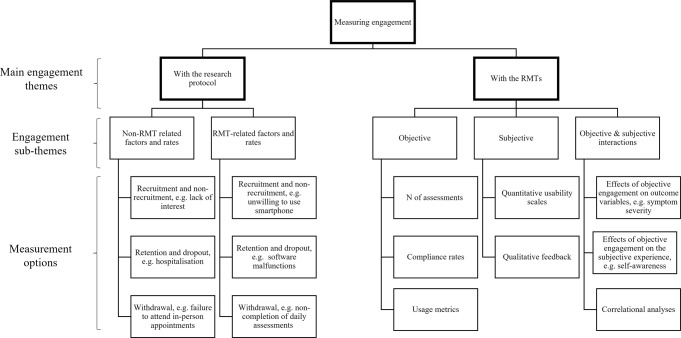
An integrative framework for the measurement of engagement with RMTs, based on the four synthetic constructs found. The main engagement themes cover ‘engagement with the research protocol’ and ‘engagement with RMTs’. Further engagement sub-themes correspond to ways in which engagement can be conceptualised within each of the two main themes. The third section outlines several available options for measurement within each sub-theme.

The synthetic constructs for measuring engagement were combined to develop an overarching synthetic argument: an integrative framework for measuring engagement with RMTs. This is depicted in Fig. 2. Definitions were considered too heterogenous to include in the framework, and instead recommendations are included within the discussion section of this review.

## Discussion

### Principal Findings

Understanding how engagement is reported in the RMT literature is imperative to ensure reproducibility in the field, to allow studies to build upon results of previous work, and to implement findings in real world settings. The first and second aims of this review were to explore the current state of defining and measuring engagement by using both quantitative and CIS data synthesis methods. The majority of papers reported on engagement in some form. However, these represented a large range of incoherent and often unjustified measures. Many studies employed several measures in one paper, resulting in a lack of distinction between measure types, explanation of why some were chosen over others, and understanding of what conclusions might be drawn from the engagement findings. A much lower proportion of studies included a corresponding definition of engagement, including many of those which described engagement as a main aim of the study. Where engagement was defined, concepts and phrases were interchangeable across papers. Indeed, even across papers with similar engagement definitions, such as ‘feasibility’, cut-offs used to measure RMT compliance differed hugely. Thus, though there is potential for the field to evaluate engagement, a lack of standardised reporting is impeding progress.

The third aim of this review was to present recommendations for the standardisation of future work. The first step towards this has been to provide clarity on the engagement measures that are currently used in the literature. The integrative framework depicted in Fig. 2 is split into two core themes of engagement measures: engagement with the research protocol and engagement with RMTs themselves. This distinction is important given that many studies did not differentiate between these concepts when reporting on engagement; using correlates of dropout from RMT studies as a proxy for gauging wider interest in RMT implementation is only relevant if the reasons for dropout were specific to the RMT aspects of the study. The engagement with RMTs section is further split into objective and subjective measurements. Interestingly, a wide range of measures were used here, but very few studies acknowledged a distinction between objective and subjective engagement or the possible interactions between the two. Under each section of the framework lies a series of options for measuring each engagement type. This framework should aid in the classification and selection of measurements when reporting on an RMT study.

**Fig. 3:**
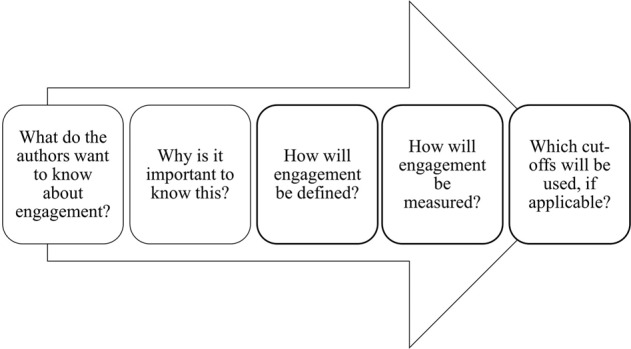
Implementing the reporting of engagement into the study design process for RMT studies. Authors are encouraged to conceptualise and define key measurement strategies for engagement during the study development phase.

The second step towards standardising future work lies in establishing ‘best practice’ guidelines for reporting on engagement. This review has highlighted several drawbacks in the current literature: a dearth of clearly conceptualised engagement definitions and corresponding measures, and, as a result, an inability to make concrete conclusions on the extent of engagement in the field. Figure 3 depicts how the process of assessing engagement with RMTs should begin as early as study development. Authors designing RMT studies are encouraged to explore the reasons for examining engagement, e.g., understanding the feasibility of using a symptom tracking app, and why it is important to know this, e.g., in order to understand the extent of data collection that could be expected with clinical implementation, or to inform approaches to missing data. Authors should then pre-define a definition, measurement(s) and applicable cut-offs to be used in answering this question. Such data could be reported in the main paper or a subsequent engagement paper. Following these guidelines could provide the necessary foundations for reproducible results in the field.

### Links with previous work

To our knowledge, this is the first review that has explored the extent of engagement reporting across the entirety of the RMT literature. This work complements and expands on previous reviews that have found heterogeneity in reporting of RMT studies^22–24^. It also might provide explanation as to why previous studies have found a wide range of dropout rates^20,21^. A lack of provision of engagement definitions in this work reflects the findings of Sieverink and colleagues in the eHealth literature^27^; a small minority of studies included an operationalisation of the engagement measure used, making comparisons difficult at best and futile at worst.

A key link to previous work is the parallel between the objective and subjective engagement concepts highlighted in Perski and colleagues’ work with DBCIs^28^. Both the definition and measurement synthetic constructs found in this review loosely map on to this distinction, with two exceptions. First, subjective engagement with RMTs is largely focused on the usability or utility of the technology, or on the subjective effects of tracking symptoms, e.g., self-awareness of symptoms, feeling monitored by a ‘safety net’. This is in comparison to the typical measures of ‘flow’ or immersion that are seen as a mechanism of action towards behaviour change in DBCIs. Second, engagement with the research protocol is a novel finding that has not been acknowledged in the DBCI literature. This is likely owing to the simultaneous use of RMTs as both a method for data collection and a tool for symptom self-management. Such differences warrant the exploration of the RMT field in its own right, as separate from DBCI or general eHealth literature.

### Strengths and limitations

This review was deliberately extensive in nature, including papers spanning physical and mental health conditions and aRMT or multiparametric RMT measures. It did not exclude papers for not reporting on engagement. This allowed for an overview of the scope of reporting across the field, in contrast to previous reviews which pre-defined engagement for the purpose of inclusion^20,22,27,88^. However, one limitation of such a broad focus is the lack of sub-typing by condition, journal type, RMT type or RMT purpose. For example, objective RMT engagement might be more important to consider in studies using RMTs for research purposes, whereas subjective RMT engagement might be more insightful for RMTs implemented into clinical practice. A specific focus on pRMT studies might also uncover additional measures of objective and subjective engagement. A second limitation is the decision to include secondary analyses papers in data synthesis. Six of the included papers reviewed analyses from the MONARCA I trial^43,45,46,51,58,89^, and a subsequent 5 on the MONARCA II trial^64,66,85,87,90^. This was justified given the tendency to report on feasibility in standalone papers, however, may have resulted in an over-representation of definitions or measures chosen by these authors. It might also be the case that papers reporting on the same dataset publish multiple analysis papers but only one engagement paper, resulting in a skew towards measures of engagement with the research protocol in these findings. It should also be considered that the searches for this review were undertaken in July 2020. However, the authors have no reason to believe that engagement reporting standards have radically changed between this date and publication of this manuscript.

### Implications for future work and conclusions

This review provides the foundation for future RMT studies to define, measure and report on engagement in a standardised and reproducible way. Authors should use both the engagement reporting process (Fig. 3) and the integrative framework (Fig. 2) as a basis for assessing engagement. At the same time, further work should be undertaken to understand how engagement reporting might differ by condition, journal type, RMT type and RMT purpose. Such findings will build on the proposed framework, making it applicable to a wider range of RMT work as the field continues to grow.

This review suggests that where authors are generally interested in measuring engagement with RMTs, the primary emphasis is placed on engaging with the research protocol or objectively with the RMTs themselves. This is logical for two reasons. First, the CONSORT-EHEALTH guidelines^25^ focus mainly on reporting of engagement with the research protocol, e.g., participant attrition, and technology process outcomes, e.g., metrics of use. Second, these types of engagement directly impact on missing data and resulting statistical analyses. However, a deeper exploration of subjective engagement with RMTs might aid further understanding. A handful of papers in this review have already examined the effects of monitoring symptoms remotely on both clinical outcome variables and feelings of self-awareness and safety. A few have also begun to acknowledge the interaction between RMT usability and future use. Future work should focus on uncovering the links between objective and subjective RMT engagement, which could have huge implications for understanding how and why users engage with these technologies for research, clinical practice, or self-management.

To conclude, the current review provides an exploration of the current state of engagement reporting in studies which use RMTs for symptom tracking in physical or mental health conditions. Where there is clearly interest, the growth of the field is currently impeded by a lack of engagement definitions, incoherent measures, and an inability to compare findings. Recommendations for the standardisation of reporting guidelines and the integration of engagement definitions and measures into the study design process have been put forward. In extending existing reporting guidelines, engagement with RMTs should also be considered as distinct from general eHealth interventions.

Future work should aim to contribute to the ongoing framework that has been provided, moving towards a unified understanding of the impact of engagement in this field, and what can be done to promote it.

## Methods

### Design

Following the Preferred Reporting Items for Systematic Reviews and Meta-analyses (PRISMA) guidelines^91^, we report on the development of the search strategy, exclusion criteria, and selection and data extraction process for this systematic review. Data synthesis was informed by critical interpretive synthesis (CIS)^92^. CIS is an analysis technique that combines both quantitative and qualitative data, and promotes an inductive approach to the development of a theoretical framework from the available literature^93^. There is currently a lack of understanding of reporting of engagement in RMTs, thus CIS was deemed an appropriate method. CIS has previously been used in reviews of engagement with eHealth^28,92^. This review has been registered on PROSPERO [CRD42020192652].

### Information sources & search strategy

A systematic search of the following seven databases was conducted in July 2020: Embase, MEDLINE and PsycINFO (all via Ovid), PubMed, IEEE Xplore, Web of Science Core Collection (comprising Science Citation Index Expanded and Social Sciences Citation Index), and Cochrane Central Register of Controlled Trials (CENTRAL). No date limits were imposed on the searches. Search terms included a combination of synonyms of i) symptom monitoring (e.g., self-monitor*), ii) remote measurement technology (e.g., smartphone app*), iii) health disorder (e.g., chronic disease*), and (iv) core symptomatology across physical and mental health conditions (mood, depress*, pain, fatigue, mobility) using the Boolean operator ‘AND’. Terms were searched for as free text in the title, abstract, or full text if available, using subject headings where appropriate (see Supplementary Note 1).

### Eligibility criteria

Papers were included if they met the following criteria: 1) a peer-reviewed, full-text publication that observes the use of aRMT or aRMT and pRMT for the purpose of symptom monitoring for a physical or mental health condition, 2) participants of 18+ years recruited on the basis of having a physical or mental health condition (clinical cohorts with diagnosed disorders or non-clinical samples with validated measures), and 3) an RMT smartphone app that prompts users to track symptoms at least three times during the study period (in order to capture at least one time point in between enrolment and study end). Studies with or without comparison groups were included. Papers were excluded for the following reasons:

- Not written in English, Spanish, or German (languages spoken by co-authors);
- A sole focus on *pre-implementation* of RMT use, e.g., purely qualitative user-experience workshops;
- Description of an RMT system without actual observed data;
- General population cohorts, e.g., fitness tracking, wellbeing studies in non-clinical samples;
- RMT properties: use of a generic symptom monitoring app not developed specifically for the research on which the paper reports, e.g., PsyMate^TM^^94^, only pRMT;
- Symptom tracking properties: data inputted through a web-link or SMS, non-prompted, user-initiated symptom monitoring only.

Papers reporting secondary analyses were included, given that they have the potential to report on engagement information that might not have been presented in the initial paper^29^. General wellbeing or fitness studies were excluded as users might have different motivations to engage than if the symptom tracking was linked with specific disease management. Generic apps were excluded on the assumption that they might carry pre-defined engagement metrics. Engagement reporting was not included in the search strategy or eligibility criteria in order to evaluate the consistency of reporting across the literature^29^. No papers were excluded on the basis of quality, in order to describe the overall state of the research field^93^.

### Study selection process

Papers identified through the search process were merged using EndNote 20^95^. Duplicate records were removed. Two pairs of reviewers independently completed the title and abstract screening stage, whereby each record was screened by at least two reviewers. Two reviewers (KMW and CW) completed the full text screening stage. Any disagreements at each stage were resolved through discussion or by consulting a third reviewer.

### Data extraction and management

A data extraction form was developed and refined, informed by recent systematic reviews on engagement in eHealth^28,29^ and a preliminary review of included papers. Due to the large volume of eligible studies, three team members performed data extraction; KMW independently checked the extraction data for accuracy. For each paper, data were extracted on i) study characteristics, ii) symptom monitoring, and iii) engagement reporting. ‘RMT use’ reflected the main aim of the RMT and was coded as (1) self-management (used to manage a condition by the individual), (2) clinical support (used in conjunction with clinical care), or (3) research (asked to use as part of a research study only). Engagement reporting was split into definition, measurement and reporting sections. For the purpose of data extraction, ‘engagement’ was loosely defined as any definition or measure pertaining to how or why participants interacted with any element of the study. No pre-determined codes were enforced onto the engagement data, in keeping with the inductive approach of CIS.

### Quality appraisal

No papers were excluded on the basis of quality. Following the principles of CIS, the process of data synthesis itself represents a critique of the literature and interpretations of credibility or methodological standards^92^. The data synthesis in this review therefore treats the literature as an ‘object of scrutiny in its own right’^92^, acting as a form of quality appraisal.

### Synthesis method

The ultimate goal of CIS is the formulation of one or more *synthesising arguments* that integrate evidence from *synthetic constructs* in the literature into a coherent theoretical framework^92^. A detailed process for conducting CIS can be found in^92^ and^28^. We took the following steps in synthesising the qualitative constructs of engagement definition and measurement:

1. Text identified in the ‘engagement reporting’ stage of data extraction for each individual paper was coded using nVivo software^96^. The specific research aims were used to guide the coding frame.
2. Codes were reviewed to develop *synthetic constructs*, whereby similar codes were grouped together to form themes.
3. Each paper was retrospectively categorised on the presence of each synthetic construct. This gave the opportunity to present a complementary quantitative overview of the data.
4. An overarching *synthesising argument* was developed (i.e., an integrative framework for reporting on engagement with RMTs) by combining all synthetic constructs.
5. The synthesising argument was refined through discussion between co-authors.

### Reporting summary

Further information on research design is available in the Nature Research Reporting Summary linked to this article.

## Supplementary information

Reporting Summary

Supplementary Information

## Data Availability

The data that support the findings of this study are available from the corresponding author upon reasonable request.

## References

[1] Matcham F (2019). Remote assessment of disease and relapse in major depressive disorder (RADAR-MDD): A multi-centre prospective cohort study protocol 11 Medical and Health Sciences 1103 Clinical Sciences 11 Medical and Health Sciences 1117 Public Health and Health Services. BMC Psychiatry.

[2] Naslund JA, Marsch LA, McHugo GJ, Bartels SJ (2015). Emerging mHealth and eHealth interventions for serious mental illness: a review of the literature. J. Ment. Health.

[3] Yuezhou, et al. Predicting depressive symptom severity through individuals’ nearby bluetooth device count data collected by mobile phones: preliminary longitudinal study. *JMIR Mhealth Uhealth* **9**, e29840 https://mhealth.jmir.org/2021/7/e29840 (2021). 10.2196/29840 PMC8367113 34328441

[4] Zhang Y (2021). Relationship Between Major Depression Symptom Severity And Sleep Collected Using A Wristband Wearable Device: Multicenter Longitudinal Observational Study. JMIR Mhealth Uhealth.

[5] Sun S (2020). Using smartphones and wearable devices to monitor behavioral changes during COVID-19. J. Med Internet Res.

[6] Myers DR, Weiss A, Rollins MR, Lam WA (2017). Towards remote assessment and screening of acute abdominal pain using only a smartphone with native accelerometers. Sci. Rep..

[7] Burchert S, Kerber A, Zimmermann J, Knaevelsrud C (2021). Screening accuracy of a 14-day smartphone ambulatory assessment of depression symptoms and mood dynamics in a general population sample: Comparison with the PHQ-9 depression screening. PLoS One.

[8] Pombo N, Garcia N, Bousson K, Spinsante S, Chorbev I (2016). Pain assessment-can it be done with a computerised system? A systematic review and meta-analysis. Int. J. Environ. Res. Public Health.

[9] Jones M, Johnston D (2011). Understanding phenomena in the real world: The case for real time data collection in health services research. J. Health Serv. Res. Policy.

[10] Moshe I (2021). Predicting symptoms of depression and anxiety using smartphone and wearable data. Front. Psychiatry.

[11] Liao Y, Thompson C, Peterson S, Mandrola J, Beg MS (2019). The future of wearable technologies and remote monitoring in health care. Am. Soc. Clin. Oncol. Educ. book. Am. Soc. Clin. Oncol. Annu. Meet..

[12] O’Brien HL, Toms EG (2008). What is user engagement? A conceptual framework for defining user engagement with technology. J. Am. Soc. Inf. Sci. Technol..

[13] Teague S (2018). Retention strategies in longitudinal cohort studies: A systematic review and meta-analysis. BMC Med. Res. Methodol..

[14] Druce KL, Dixon WG, McBeth J (2019). Maximizing engagement in mobile health studies: lessons learned and future directions. Rheum. Dis. Clin. North Am..

[15] Simblett S (2019). Barriers to and facilitators of engagement with mhealth technology for remote measurement and management of depression: qualitative analysis. JMIR mHealth uHealth.

[16] Simblett SK (2019). Engaging across dimensions of diversity: A cross-national perspective on mHealth tools for managing relapsing remitting and progressive multiple sclerosis. Mult. Scler. Relat. Disord..

[17] Simblett SK (2019). Patient perspectives on the acceptability of mHealth technology for remote measurement and management of epilepsy: A qualitative analysis. Epilepsy Behav..

[18] White KM (2021). Remote measurement in rheumatoid arthritis: qualitative analysis of patient perspectives. JMIR Form. Res.

[19] Vanderboom CE, Vincent A, Luedtke CA, Rhudy LM, Bowles KH (2014). Feasibility of interactive technology for symptom monitoring in patients with fibromyalgia. Pain. Manag. Nurs..

[20] Girolamo, G. et al. The acceptability of real‐time health monitoring among community participants with depression: A systematic review and meta‐analysis of the literature. Depress. Anxiety da.23023 10.1002/da.23023 (2020).

[21] Torous J, Lipschitz J, Ng M, Firth J (2020). Dropout rates in clinical trials of smartphone apps for depressive symptoms: A systematic review and meta-analysis. J. Affect. Disord..

[22] Simblett, S. et al. Barriers to and facilitators of engagement with remote measurement technology for managing health: Systematic review and content analysis of findings. *J. Med. Internet Res*. **20**, (2018). 10.2196/10480 PMC6062692 30001997

[23] Dogan, E., Sander, C., Wagner, X., Hegerl, U. & Kohls, E. Smartphone-based monitoring of objective and subjective data in affective disorders: Where are we and where are we going? Systematic review. *J. Med. Internet Res*. **19**, 10.2196/jmir.7006 (2017). 10.2196/jmir.7006 PMC5547249 28739561

[24] de Angel, V. et al. Digital health tools for the passive monitoring of depression: a systematic review of methodsTitle. *npj Digit. Med*. **5**, 3 10.1038/s41746-021-00548-8 (2022). 10.1038/s41746-021-00548-8 PMC8752685 35017634

[25] Eysenbach, G. & Group, C. CONSORT ‐ EHEALTH checklist (V. 1. 6. 1): Information to include when reporting ehealth / mhealth trials (web ‐ based / Internet ‐ based intervention and decision aids, but also social media, serious games, DVDs, mobile applications, certain te. 1–13 (2011).

[26] von Elm E (2008). The Strengthening the Reporting of Observational Studies in Epidemiology (STROBE) statement: guidelines for reporting observational studies. J. Clin. Epidemiol..

[27] Sieverink F, Kelders SM, Gemert-Pijnen V (2017). Clarifying the concept of adherence to ehealth technology: Systematic review on when usage becomes adherence. J. Med. Internet Res..

[28] Perski O, Blandford A, West R, Michie S (2017). Conceptualising engagement with digital behaviour change interventions: a systematic review using principles from critical interpretive synthesis. Transl. Behav. Med..

[29] Molloy A, Anderson PL (2021). Engagement with mobile health interventions for depression: A systematic review. Internet Inter..

[30] Li H (2019). Use of ecological momentary assessment to detect variability in mood, sleep and stress in bipolar disorder. BMC Res. Notes.

[31] Juengst SB, Terhorst L, Kew CL, Wagner AK (2019). Variability in daily self-reported emotional symptoms and fatigue measured over eight weeks in community dwelling individuals with traumatic brain injury. Brain Inj..

[32] Greer JA (2020). Randomized trial of a smartphone mobile app to improve symptoms and adherence to oral therapy for cancer. J. Natl Compr. Cancer Netw..

[33] Jamison RN, Jurcik DC, Edwards RR, Huang C-CC, Ross EL (2017). A pilot comparison of a smartphone app with or without 2-way messaging among chronic pain patients: who benefits from a pain app?. Clin. J. Pain..

[34] Schwartz S, Schultz S, Reider A, Saunders EFH (2016). Daily mood monitoring of symptoms using smartphones in bipolar disorder: A pilot study assessing the feasibility of ecological momentary assessment. J. Affect. Disord..

[35] Bove, R. et al. Evaluating more naturalistic outcome measures. *Neurol. Neuroimmunol. NeuroInflammation* **2**, 10.1212/NXI.0000000000000162 (2015). 10.1212/NXI.0000000000000162 PMC4608760 26516627

[36] Zia J (2016). Feasibility and usability pilot study of a novel irritable bowel syndrome food and gastrointestinal symptom journal smartphone app. Clin. Transl. Gastroenterol..

[37] Jamison RN, Mei A, Ross EL (2018). Longitudinal trial of a smartphone pain application for chronic pain patients: Predictors of compliance and satisfaction. J. Telemed. Telecare.

[38] Selter A (2018). An mhealth app for self-management of chronic lower back pain (Limbr): Pilot study. JMIR mHealth uHealth.

[39] Rijsbergen M (2020). Mobile e-diary application facilitates the monitoring of patient-reported outcomes and a high treatment adherence for clinical trials in dermatology. J. Eur. Acad. Dermatol. Venereol..

[40] Van Til K, McInnis MG, Cochran A (2020). A comparative study of engagement in mobile and wearable health monitoring for bipolar disorder. Bipolar Disord..

[41] Anguera JA, Jordan JT, Castaneda D, Gazzaley A, Areán PA (2016). Conducting a fully mobile and randomised clinical trial for depression: Access, engagement and expense. BMJ Innov..

[42] Bauer AM (2018). Acceptability of mHealth augmentation of Collaborative Care: A mixed methods pilot study. Gen. Hosp. Psychiatry.

[43] Faurholt-Jepsen M (2015). Daily electronic self-monitoring in bipolar disorder using smartphones - The MONARCA i trial: A randomized, placebo-controlled, single-blind, parallel group trial. Psychol. Med..

[44] Faurholt-Jepsen M (2019). Smartphone-based self-monitoring in bipolar disorder: evaluation of usability and feasibility of two systems. Int. J. Bipolar Disord..

[45] Faurholt-Jepsen M (2016). Voice analysis as an objective state marker in bipolar disorder. Transl. Psychiatry.

[46] Stanislaus S (2020). Mood instability in patients with newly diagnosed bipolar disorder, unaffected relatives, and healthy control individuals measured daily using smartphones. J. Affect. Disord..

[47] Gustavell T, Sundberg K, Segersvärd R, Wengström Y, Langius-Eklöf A (2019). Decreased symptom burden following surgery due to support from an interactive app for symptom management for patients with pancreatic and periampullary cancer. Acta Oncol. (Madr.).

[48] Niendam TA (2018). Enhancing early psychosis treatment using smartphone technology: A longitudinal feasibility and validity study. J. Psychiatr. Res..

[49] Jamison RN, Mei A, Edwards RR, Ross EL (2018). Efficacy of vibrating gloves for chronic hand pain due to osteoarthritis. Pain. Med. (U. S.).

[50] Crouthamel M (2018). Using a researchkit smartphone app to collect rheumatoid arthritis symptoms from real-world participants: Feasibility study. JMIR mHealth uHealth.

[51] Faurholt-Jepsen M (2016). Behavioral activities collected through smartphones and the association with illness activity in bipolar disorder. Int. J. Methods Psychiatr. Res..

[52] Hung S (2016). Smartphone-based ecological momentary assessment for Chinese patients with depression: An exploratory study in Taiwan. Asian J. Psychiatr..

[53] Rodriguez Hermosa JL (2020). Compliance and utility of a smartphone app for the detection of exacerbations in patients with chronic obstructive pulmonary disease: cohort study. JMIR mHealth uHealth.

[54] Buck B (2019). Capturing behavioral indicators of persecutory ideation using mobile technology. J. Psychiatr. Res..

[55] Moukaddam N, Truong A, Cao J, Shah A, Sabharwal A (2019). Findings from a trial of the smartphone and online usage-based evaluation for depression (SOLVD) application: What do apps really tell us about patients with depression? concordance between app-generated data and standard psychiatric questionnaires for de. J. Psychiatr. Pr..

[56] Broderick, J. E. et al. Patient reported outcomes can improve performance status assessment: a pilot study. *J. Patient-Reported Outcomes* **3**, 10.1186/s41687-019-0136-z (2019). 10.1186/s41687-019-0136-z PMC6635569 31313047

[57] Lenaert B, Neijmeijer M, van Kampen N, van Heugten C, Ponds R (2020). Poststroke fatigue and daily activity patterns during outpatient rehabilitation: an experience sampling method study. Arch. Phys. Med. Rehabil..

[58] Faurholt-Jepsen M (2014). Smartphone data as objective measures of bipolar disorder symptoms. Psychiatry Res.

[59] Kim J (2016). Depression screening using daily mental-health ratings from a smartphone application for breast cancer patients. J. Med. Internet Res..

[60] Band R, Barrowclough C, Caldwell K, Emsley R, Wearden A (2017). Activity patterns in response to symptoms in patients being treated for chronic fatigue syndrome: An experience sampling methodology study. J. Div. Heal. Psychol. Am. Psychol. Assoc..

[61] Probst T (2017). Does tinnitus depend on time-of-day? An ecological momentary assessment study with the ‘TrackYourTinnitus’ application. Front. Aging Neurosci..

[62] Kauer SD (2012). Self-monitoring using mobile phones in the early stages of adolescent depression: Randomized controlled trial. J. Med. Internet Res..

[63] Carpenter RW, Lane SP, Bruehl S, Trull TJ (2019). Concurrent and lagged associations of prescription opioid use with pain and negative affect in the daily lives of chronic pain patients. J. Consult. Clin. Psychol..

[64] Faurholt-Jepsen M (2019). Is smartphone-based mood instability associated with stress, quality of life, and functioning in bipolar disorder?. Bipolar Disord..

[65] Ireland D, Andrews N (2019). Pain ROADMAP: a mobile platform to support activity pacing for chronic pain. Stud. Health Technol. Inform..

[66] Faurholt-Jepsen, M. et al. Differences in mood instability in patients with bipolar disorder type I and II: a smartphone-based study. *Int. J. Bipolar Disord*. **7**, 10.1186/s40345-019-0141-4 (2019). 10.1186/s40345-019-0141-4 PMC6355891 30706154

[67] Reid SC (2011). A mobile phone application for the assessment and management of youth mental health problems in primary care: A randomised controlled trial. BMC Fam. Pract..

[68] Corden ME (2016). MedLink: A mobile intervention to improve medication adherence and processes of care for treatment of depression in general medicine. Digit. Heal.

[69] Lin W-C, Burke L, Schlenk EA, Yeh CH (2019). Use of an ecological momentary assessment application to assess the effects of auricular point acupressure for chronic low back pain. Comput. Inform. Nurs..

[70] Yang YS, Ryu GW, Choi M (2019). Factors associated with daily completion rates in a smartphone-based ecological momentary assessment study. Healthc. Inform. Res..

[71] Torous J (2015). Utilizing a personal smartphone custom app to assess the patient health questionnaire-9 (phq-9) depressive symptoms in patients with major depressive disorder. JMIR Ment. Heal.

[72] Suso-Ribera C (2018). Validity, reliability, feasibility, and usefulness of pain monitor. Clin. J. Pain..

[73] Wu JQ, Cronin-Golomb A (2020). Temporal associations between sleep and daytime functioning in Parkinson’s disease: a smartphone-based ecological momentary assessment. Behav. Sleep. Med..

[74] Beiwinkel, T. et al. Using smartphones to monitor bipolar disorder symptoms: A pilot study. *JMIR Ment. Heal*. **3**, 10.2196/mental.4560 (2016). 10.2196/mental.4560 PMC4720836 26740354

[75] Ben-Zeev D (2014). Feasibility, acceptability, and preliminary efficacy of a smartphone intervention for schizophrenia. Schizophr. Bull..

[76] Prada P (2017). EMOTEO: A Smartphone Application for Monitoring and Reducing Aversive Tension in Borderline Personality Disorder Patients, a Pilot Study. Perspect. Psychiatr. Care.

[77] Sengupta, A., Beckie, T., Dutta, K., Dey, A. & Chellappan, S. A mobile health intervention system for women with coronary heart disease: usability study. *JMIR Form. Res*. **4**, (2020). 10.2196/16420 PMC7301266 32348270

[78] Gustavell T, Sundberg K, Langius-Eklöf A (2020). Using an interactive app for symptom reporting and management following pancreatic cancer surgery to facilitate person-centered care: Descriptive study. JMIR mHealth uHealth.

[79] Reid SC (2013). A mobile phone application for the assessment and management of youth mental health problems in primary care: Health service outcomes from a randomised controlled trial of mobiletype. BMC Fam. Pract..

[80] Suso-Ribera C (2018). Validity, reliability, feasibility, and usefulness of pain monitor: a multidimensional smartphone app for daily monitoring of adults with heterogenous chronic pain. Clin. J. Pain..

[81] Bandarian-Balooch S, Martin PR, McNally B, Brunelli A, Mackenzie S (2017). Electronic-diary for recording headaches, triggers, and medication use: development and evaluation. Headache.

[82] Mohr DC (2015). MedLink: a mobile intervention to address failure points in the treatment of depression in general medicine. Int. Conf. Pervasive Comput. Technol. Health. [Proc.]. Int. Conf. Pervasive Comput. Technol. Health..

[83] Hidalgo-Mazzei D (2016). Psychoeducation in bipolar disorder with a SIMPLe smartphone application: Feasibility, acceptability and satisfaction. J. Affect. Disord..

[84] Ross EL, Jamison RN, Nicholls L, Perry BM, Nolen KD (2020). Clinical integration of a smartphone app for patients with chronic pain: retrospective analysis of predictors of benefits and patient engagement between clinic visits. J. Med. Internet Res..

[85] Faurholt-Jepsen M (2020). The effect of smartphone-based monitoring on illness activity in bipolar disorder: The MONARCA II randomized controlled single-blinded trial. Psychol. Med..

[86] Shafran R (2019). Translating the intention to seek treatment into action: does symptom monitoring make a difference? results from a randomized controlled trial. Behav. Cogn. Psychother..

[87] Busk J (2020). Forecasting mood in bipolar disorder from smartphone self-assessments: hierarchical Bayesian approach. JMIR mHealth uHealth.

[88] Barello, S. et al. eHealth for patient engagement: A Systematic Review. *Front. Psychol*. **6**, 10.3389/fpsyg.2015.02013 (2016). 10.3389/fpsyg.2015.02013 PMC4705444 26779108

[89] Faurholt-Jepsen M (2015). Mood instability in bipolar disorder type I versus type II-continuous daily electronic self-monitoring of illness activity using smartphones. J. Affect. Disord..

[90] Busk, J. et al. Daily estimates of clinical severity of symptoms in bipolar disorder from smartphone-based self-assessments. *Transl. Psychiatry* **10**, 10.1038/s41398-020-00867-6 (2020). 10.1038/s41398-020-00867-6 PMC7303106 32555144

[91] Page, M. J. et al. The PRISMA 2020 statement: an updated guideline for reporting systematic reviews. *BMJ* **372** (2021). 10.1136/bmj.n71 PMC8005924 33782057

[92] Dixon-Woods M (2006). Conducting a critical interpretive synthesis of the literature on access to healthcare by vulnerable groups. BMC Med. Res. Methodol..

[93] Depraetere, J., Vandeviver, C., Keygnaert, I. & Beken, T. V. The critical interpretive synthesis: an assessment of reporting practices. *Int. J. Soc. Res. Methodol*. 10.1080/13645579.2020.1799637 (2020).

[94] Maastricht University. PsyMate. https://www.psymate.eu/.

[95] Team, T. E. EndNote. (2013).

[96] QSR International Pty Ltd. nVivo. (2020).

