# Supplementary material for: A systematic review of engagement reporting in remote measurement studies for health symptom tracking
Source: NPJ Digit Med. 2022 Jun 29;5:82. doi: 10.1038/s41746-022-00624-7 (PMC9242990; doi:10.1038/s41746-022-00624-7)
Supplement: Supplementary file 1 — Reporting Summary [file 41746_2022_624_MOESM1_ESM.pdf]

nature portfolio

Corresponding author(s): Katie M White

Last updated by author(s): May 26, 2022

Reporting Summary

Nature Portfolio wishes to improve the reproducibility of the work that we publish. This form provides structure for consistency and transparency in reporting. For further information on Nature Portfolio policies, see our Editorial Policies and the Editorial Policy Checklist.

Statistics

For all statistical analyses, confirm that the following items are present in the figure legend, table legend, main text, or Methods section.

n/a	Confirmed	
☒	☐	The exact sample size (n) for each experimental group/condition, given as a discrete number and unit of measurement
☒	☐	A statement on whether measurements were taken from distinct samples or whether the same sample was measured repeatedly
☒	☐	The statistical test(s) used AND whether they are one- or two-sided<br>*Only common tests should be described solely by name; describe more complex techniques in the Methods section.*
☒	☐	A description of all covariates tested
☒	☐	A description of any assumptions or corrections, such as tests of normality and adjustment for multiple comparisons
☒	☐	A full description of the statistical parameters including central tendency (e.g. means) or other basic estimates (e.g. regression coefficient) AND variation (e.g. standard deviation) or associated estimates of uncertainty (e.g. confidence intervals)
☒	☐	For null hypothesis testing, the test statistic (e.g. F, t, r) with confidence intervals, effect sizes, degrees of freedom and P value noted<br>*Give P values as exact values whenever suitable.*
☒	☐	For Bayesian analysis, information on the choice of priors and Markov chain Monte Carlo settings
☒	☐	For hierarchical and complex designs, identification of the appropriate level for tests and full reporting of outcomes
☒	☐	Estimates of effect sizes (e.g. Cohen's d, Pearson's r), indicating how they were calculated

Our web collection on statistics for biologists contains articles on many of the points above.

Software and code

Policy information about availability of computer code

Data collection: N/A

Data analysis: N/A

For manuscripts utilizing custom algorithms or software that are central to the research but not yet described in published literature, software must be made available to editors and reviewers. We strongly encourage code deposition in a community repository (e.g. GitHub). See the Nature Portfolio guidelines for submitting code & software for further information.

Data

Policy information about availability of data

All manuscripts must include a data availability statement. This statement should provide the following information, where applicable:

- Accession codes, unique identifiers, or web links for publicly available datasets
- A description of any restrictions on data availability
- For clinical datasets or third party data, please ensure that the statement adheres to our policy

The data that support the findings of this study are available from the corresponding author upon reasonable request.

Field-specific reporting

Please select the one below that is the best fit for your research. If you are not sure, read the appropriate sections before making your selection.

☐ Life sciences ☒ Behavioural & social sciences ☐ Ecological, evolutionary & environmental sciences

For a reference copy of the document with all sections, see nature.com/documents/nr-reporting-summary-flat.pdf

Behavioural & social sciences study design

All studies must disclose on these points even when the disclosure is negative.

Study description	This review aimed to synthesise the RMT literature to identify how and to what extent engagement is defined, measured, and reported, and to present recommendations for the standardisation of future work.
Research sample	Seven databases (Embase, MEDLINE and PsycINFO (via Ovid), PubMed, IEEE Xplore, Web of Science, and Cochrane Central Register of Controlled Trials) were searched in July 2020 for papers using RMT apps for symptom monitoring in adults with a health condition, prompting users to track at least three times during the study period. Data were synthesised using critical interpretive synthesis. A total of 76 papers met the inclusion criteria.
Sampling strategy	Seven databases (Embase, MEDLINE and PsycINFO (via Ovid), PubMed, IEEE Xplore, Web of Science, and Cochrane Central Register of Controlled Trials) were searched in July 2020 for papers using RMT apps for symptom monitoring in adults with a health condition, prompting users to track at least three times during the study period. Data were synthesised using critical interpretive synthesis. A total of 76 papers met the inclusion criteria.
Data collection	Seven databases (Embase, MEDLINE and PsycINFO (via Ovid), PubMed, IEEE Xplore, Web of Science, and Cochrane Central Register of Controlled Trials) were searched in July 2020 for papers using RMT apps for symptom monitoring in adults with a health condition, prompting users to track at least three times during the study period. Data were synthesised using critical interpretive synthesis. A total of 76 papers met the inclusion criteria.
Timing	July 2020
Data exclusions	Papers were excluded for the following reasons:<br><br>Not written in English, Spanish, or German (languages spoken by co-authors);<br><br>A sole focus on pre-implementation of RMT use, e.g. purely qualitative user-experience workshops;<br><br>Description of an RMT system without actual observed data;<br><br>General population cohorts, e.g. fitness tracking, wellbeing studies in non-clinical samples;<br><br>RMT properties: use of a generic symptom monitoring app not developed specifically for the research on which the paper reports, e.g. PsyMateTM 94, only pRMT;<br><br>Symptom tracking properties: data inputted through a web-link or SMS, non-prompted, user-initiated symptom monitoring only.
Non-participation	N/A
Randomization	N/A

Reporting for specific materials, systems and methods

We require information from authors about some types of materials, experimental systems and methods used in many studies. Here, indicate whether each material, system or method listed is relevant to your study. If you are not sure if a list item applies to your research, read the appropriate section before selecting a response.

Materials & experimental systems

n/a	Involved in the study	
☒	☐	Antibodies
☒	☐	Eukaryotic cell lines
☒	☐	Palaeontology and archaeology
☒	☐	Animals and other organisms
☒	☐	Human research participants
☒	☐	Clinical data
☒	☐	Dual use research of concern

Methods

n/a	Involved in the study	
☒	☐	ChIP-seq
☒	☐	Flow cytometry
☒	☐	MRI-based neuroimaging
