# Supplementary material for: A systematic review of engagement reporting in remote measurement studies for health symptom tracking
Source: NPJ Digit Med. 2022 Jun 29;5:82. doi: 10.1038/s41746-022-00624-7 (PMC9242990; doi:10.1038/s41746-022-00624-7)
Supplement: Supplementary file 2 — Supplementary Information [file 41746_2022_624_MOESM2_ESM.pdf]

Supplementary Note 1. Search strategies by database.

PubMed (1996- July 2020)
((((((((((((((((ambulatory monitoring[mh]) OR (ecological momentary assessment[mh])) OR ("symptom monitoring"[Title/Abstract])) OR ("self-monitor*"[Title/Abstract])) OR ("symptom management"[Title/Abstract])) OR ("symptom measur*"[Title/Abstract])) OR ("self-assess*"[Title/Abstract])) OR ("self-observe"[Title/Abstract])) OR (chart[Title/Abstract])) OR ("active monitoring"[Text Word])) OR ("symptom reporting"[Text Word])) OR (monitor*[Text Word])) OR ("electronic diar*"[Text Word])) OR ("daily diar*"[Text Word])) OR ("experience sampling"[Text Word])) AND (((((health [mh]) OR (mental health [mh])) OR (disease [mh])) OR (disorder[Text Word])) OR (chronic[Text Word]))) AND ((((((((((((remote sensing technology[mh:noexp]) OR (smartphone[mh])) OR (mobile applications[mh])) OR (telemedicine[mh])) OR (mobile[Title/Abstract])) OR (smartphone[Title/Abstract])) OR (app[Title/Abstract])) OR (remote[Title/Abstract])) OR (digital[Title/Abstract])) OR (wearable[Text Word])) OR (electronic[Text Word]))) AND (((((mood) OR (depress*)) OR (pain)) OR (fatigue)) OR (mobility))

Embase (via Ovid; 1974- July 2020)

1. exp ambulatory monitoring/
2. exp ecological momentary assessment/
3. "symptom monitoring".ti,ab.
4. "self-monitor*".ti,ab.
5. "symptom management".ti,ab.
6. "symptom track*".ti,ab.
7. "self-assess*".mp.
8. "active monitoring".mp.
9. "symptom reporting".mp.
10. "electronic diar*".mp.
11. "daily diar*".mp.
12. "experience sampling".mp.
13. 1 or 2 or 3 or 4 or 5 or 6 or 7 or 8 or 9 or 10 or 11 or 12
14. exp health/
15. exp mental health/
16. disorder.mp.
17. chronic.mp.
18. 14 or 15 or 16 or 17
19. exp mobile phone/
20. exp mobile application/
21. exp telehealth/
22. "mobile app*".ti,ab.
23. "smartphone app*".ti,ab.
24. app.ti,ab.
25. remote.ti,ab.
26. digital.ti,ab.
27. mobile.ti,ab.
28. wearable.mp.
29. electronic.mp.

30. 19 or 20 or 21 or 22 or 23 or 24 or 25 or 26 or 27 or 28 or 29
31. mood.mp.
32. depress*.mp.
33. pain.mp.
34. fatigue.mp.
35. mobility.mp.
36. 31 or 32 or 33 or 34 or 35
37. 13 and 18 and 30 and 36

MEDLINE (via Ovid; 1946- July 2020)

1. exp Monitoring, Ambulatory/
2. exp ecological momentary assessment/
3. "symptom monitoring".ti,ab.
4. "self-monitor*".ti,ab.
5. "symptom management".ti,ab.
6. "symptom track*".ti,ab.
7. "self-assess*".mp.
8. "active monitoring".mp.
9. "symptom reporting".mp.
10. "electronic diar*".mp.
11. "daily diar*".mp.
12. "experience sampling".mp.
13. 1 or 2 or 3 or 4 or 5 or 6 or 7 or 8 or 9 or 10 or 11 or 12
14. exp health/
15. exp mental health/
16. disorder.mp.
17. chronic.mp.
18. 14 or 15 or 16 or 17
19. exp smartphone/
20. exp mobile applications/
21. exp telemedicine/
22. "mobile app*".ti,ab.
23. "smartphone app*".ti,ab.
24. app.ti,ab.
25. remote.ti,ab.
26. digital.ti,ab.
27. mobile.ti,ab.
28. wearable.mp.
29. electronic.mp.
30. 19 or 20 or 21 or 22 or 23 or 24 or 25 or 26 or 27 or 28 or 29
31. mood.mp.
32. depress*.mp.
33. pain.mp.
34. fatigue.mp.
35. mobility.mp.
36. 31 or 32 or 33 or 34 or 35
37. 13 and 18 and 30 and 36

PsycINFO (via Ovid; 1967- July 2020)

1. exp ecological momentary assessment/
2. "ambulatory monitoring".mp.
3. "symptom monitoring".ti,ab.
4. "self-monitor*".ti,ab.
5. "symptom management".ti,ab.
6. "symptom track*".ti,ab.
7. "self-assess*".mp.
8. "active monitoring".mp.
9. "symptom reporting".mp.
10. "electronic diar*".mp.
11. "daily diar*".mp.
12. "experience sampling".mp.
13. 1 or 2 or 3 or 4 or 5 or 6 or 7 or 8 or 9 or 10 or 11 or 12
14. exp disorders/
15. exp health/
16. disorder.mp.
17. chronic.mp.
18. 14 or 15 or 16 or 17
19. exp mobile phones/
20. exp mobile applications/
21. exp telemedicine/
22. "mobile app*".ti,ab.
23. "smartphone app*".ti,ab.
24. app.ti,ab.
25. remote.ti,ab.
26. digital.ti,ab.
27. mobile.ti,ab.
28. wearable.mp.
29. electronic.mp.
30. 19 or 20 or 21 or 22 or 23 or 24 or 25 or 26 or 27 or 28 or 29
31. mood.mp.
32. depress*.mp.
33. pain.mp.
34. fatigue.mp.
35. mobility.mp.
36. 31 or 32 or 33 or 34 or 35
37. 13 and 18 and 30 and 36

IEEE Xplore (2000-July 2020)

((((((((((All Metadata:mood) OR All Metadata:depression) OR All Metadata:depressive) OR All Metadata:pain) OR All Metadata:fatigue) OR All Metadata:mobility))) AND ((((((((((((INSPEC Controlled Terms:patient monitoring) OR INSPEC Non-Controlled Terms:EMA) OR Abstract:"symptom monitoring") OR Abstract:"self-monitor") OR Abstract:"symptom management") OR Abstract:"symptom measur*") OR Abstract:"self-assess*") OR Abstract:"self-observe") OR All Metadata:"active monitoring")

OR All Metadata:"symptom reporting") OR All Metadata:"daily diar*"))) AND (((((INSPEC Controlled Terms:health care) OR INSPEC Controlled Terms:medical disorders) OR All Metadata:disorder) OR All Metadata:chronic))) AND ((((((((((((IEEE Terms:smart phones) OR INSPEC Non-Controlled Terms:smartphone app) OR INSPEC Controlled Terms:telemedicine) OR Abstract:"mobile app*") OR Abstract:"smartphone app*") OR Abstract:app) OR Abstract:remote) OR Abstract:digital) OR Abstract:mobile) OR All Metadata:wearable) OR All Metadata:electronic))

Web of Science Core Collection (1900-July 2020)

# 38	#38 AND #31<br>*Indexes=SCI-EXPANDED, SSCI, A&HCI, CPCI-S, CPCI-SSH, ESCI Timespan=All years*
# 37	#36 OR #35 OR #34 OR #33 OR #32<br>*Indexes=SCI-EXPANDED, SSCI, A&HCI, CPCI-S, CPCI-SSH, ESCI Timespan=All years*
# 36	ALL=mobility<br>*Indexes=SCI-EXPANDED, SSCI, A&HCI, CPCI-S, CPCI-SSH, ESCI Timespan=All years*
# 35	ALL=fatigue<br>*Indexes=SCI-EXPANDED, SSCI, A&HCI, CPCI-S, CPCI-SSH, ESCI Timespan=All years*
# 34	ALL=pain<br>*Indexes=SCI-EXPANDED, SSCI,*

	A&HCI, CPCI-S, CPCI-SSH, ESCI Timespan=All years
# 33	ALL=depress* *Indexes=SCI-EXPANDED, SSCI, A&HCI, CPCI-S, CPCI-SSH, ESCI Timespan=All years*
# 32	ALL=mood *Indexes=SCI-EXPANDED, SSCI, A&HCI, CPCI-S, CPCI-SSH, ESCI Timespan=All years*

# 31	#30 AND #18 AND #13 *Indexes=SCI-EXPANDED, SSCI, A&HCI, CPCI-S, CPCI-SSH, ESCI Timespan=All years*
# 30	#29 OR #28 OR #27 OR #26 OR #25 OR #24 OR #23 OR #22 OR #21 OR #20 OR #19 *Indexes=SCI-EXPANDED, SSCI, A&HCI, CPCI-S, CPCI-SSH, ESCI Timespan=All years*

# 29	ALL=electronic<br>*Indexes=SCI-EXPANDED, SSCI, A&HCI, CPCI-S, CPCI-SSH, ESCI Timespan=All years*
# 28	ALL=wearable<br>*Indexes=SCI-EXPANDED, SSCI, A&HCI, CPCI-S, CPCI-SSH, ESCI Timespan=All years*
# 27	AB=mobile<br>*Indexes=SCI-EXPANDED, SSCI, A&HCI, CPCI-S, CPCI-SSH, ESCI Timespan=All years*
# 26	AB=digital<br>*Indexes=SCI-EXPANDED, SSCI, A&HCI, CPCI-S, CPCI-SSH, ESCI Timespan=All years*
# 25	AB=remote<br>*Indexes=SCI-EXPANDED, SSCI, A&HCI, CPCI-S, CPCI-SSH, ESCI Timespan=All years*
# 24	AB=app

	Indexes=SCI-EXPANDED, SSCI, A&HCI, CPCI-S, CPCI-SSH, ESCI Timespan=All years
# 23	AB="smartphone app*" *Indexes=SCI-EXPANDED, SSCI, A&HCI, CPCI-S, CPCI-SSH, ESCI Timespan=All years*
# 22	AB="mobile app*" *Indexes=SCI-EXPANDED, SSCI, A&HCI, CPCI-S, CPCI-SSH, ESCI Timespan=All years*
# 21	ALL=telehealth *Indexes=SCI-EXPANDED, SSCI, A&HCI, CPCI-S, CPCI-SSH, ESCI Timespan=All years*
# 20	ALL="mobile phone" *Indexes=SCI-EXPANDED, SSCI, A&HCI, CPCI-S, CPCI-SSH, ESCI Timespan=All years*

# 19	ALL=smartphone<br>*Indexes=SCI-EXPANDED, SSCI, A&HCI, CPCI-S, CPCI-SSH, ESCI Timespan=All years*
# 18	#17 OR #16 OR #15 OR #14<br>*Indexes=SCI-EXPANDED, SSCI, A&HCI, CPCI-S, CPCI-SSH, ESCI Timespan=All years*
# 17	ALL=chronic<br>*Indexes=SCI-EXPANDED, SSCI, A&HCI, CPCI-S, CPCI-SSH, ESCI Timespan=All years*
# 16	ALL=disorder<br>*Indexes=SCI-EXPANDED, SSCI, A&HCI, CPCI-S, CPCI-SSH, ESCI Timespan=All years*
# 15	ALL="mental health"<br>*Indexes=SCI-EXPANDED, SSCI, A&HCI, CPCI-S, CPCI-*

	SSH, ESCI<br>*Timespan=All years*
# 14	ALL=health<br>*Indexes=SCI-EXPANDED, SSCI, A&HCI, CPCI-S, CPCI-SSH, ESCI*<br>*Timespan=All years*
# 13	#12 OR #11 OR #10 OR #9 OR #8 OR #7 OR #6 OR #5 OR #4 OR #3 OR #2 OR #1<br>*Indexes=SCI-EXPANDED, SSCI, A&HCI, CPCI-S, CPCI-SSH, ESCI*<br>*Timespan=All years*
# 12	ALL="experience sampling"<br>*Indexes=SCI-EXPANDED, SSCI, A&HCI, CPCI-S, CPCI-SSH, ESCI*<br>*Timespan=All years*
# 11	ALL="daily diar*"<br>*Indexes=SCI-EXPANDED, SSCI, A&HCI, CPCI-S, CPCI-SSH, ESCI*<br>*Timespan=All years*

# 10	ALL="electronic diar*" *Indexes=SCI-EXPANDED, SSCI, A&HCI, CPCI-S, CPCI-SSH, ESCI Timespan=All years*
# 9	ALL="symptom reporting" *Indexes=SCI-EXPANDED, SSCI, A&HCI, CPCI-S, CPCI-SSH, ESCI Timespan=All years*
# 8	ALL="active monitoring" *Indexes=SCI-EXPANDED, SSCI, A&HCI, CPCI-S, CPCI-SSH, ESCI Timespan=All years*
# 7	ALL="self-assess*" *Indexes=SCI-EXPANDED, SSCI, A&HCI, CPCI-S, CPCI-SSH, ESCI Timespan=All years*
# 6	AB="symptom track*"

	Indexes=SCI-EXPANDED, SSCI, A&HCI, CPCI-S, CPCI-SSH, ESCI Timespan=All years
# 5	AB="symptom management" *Indexes=SCI-EXPANDED, SSCI, A&HCI, CPCI-S, CPCI-SSH, ESCI Timespan=All years*
# 4	AB="self-monitor*" *Indexes=SCI-EXPANDED, SSCI, A&HCI, CPCI-S, CPCI-SSH, ESCI Timespan=All years*
# 3	AB="symptom monitoring" *Indexes=SCI-EXPANDED, SSCI, A&HCI, CPCI-S, CPCI-SSH, ESCI Timespan=All years*
# 2	ALL="ecological momentary assessment" *Indexes=SCI-EXPANDED, SSCI, A&HCI, CPCI-S, CPCI-*

	SSH, ESCI<br>*Timespan=All years*
# 1	ALL="ambulatory monitoring"<br>*Indexes=SCI-EXPANDED, SSCI, A&HCI, CPCI-S, CPCI-SSH, ESCI*<br>*Timespan=All years*

Supplementary Table 1. Characteristics of included studies.

Authors	Country	Condition	Main aims	Study design	Setting	Sample Characteristics	RMT use	RMT (aRMt only/ combined)	Duration	Engagement reporting	
										**Definition**	**Measurement**
**Anand & Anand (2019)**	US	Bipolar and Unipolar Major Depressive Disorder (MDD)	To investigate whether latent class analyses of ratings can detect mood instability among MDD and BD groups.	Prospective, observational, case control	Community	N = 40;<br>Mean age (SD) = 24.1 (NR);<br>% Female = 65	Research	aRMT only. Ginger.io Behaviour Platform app	44 weeks		X
**Anguera et al. (2016)**	US	Depression	**To determine the feasibility of conducting a fully remote RCT using smart devices in depressed adults.**	Fully mobile RCT comparing 3 different apps	Community	N = 1098;<br>Mean age (SD) = 32.9 (NR);<br>% Female = 79	Self-management	Combined. 3 treatment apps: EVO, PST, HT Assessment apps: Ginger.io (aRMT & pRMT), ACE cognitive assessment	12 weeks	X	X
**Band et al. (2016)**	UK	Chronic Fatigue Syndrome	To examine the relationship between significant other responses and patient outcomes with CFS within the context of daily life.	Prospective, ESM	Clinical	N = 46;<br>Mean age (SD) = 35.5 (13.96);<br>% Female = 87	Research	aRMT only. CFS-specific version of ClinTouch app	6 days		X
**Band et al. (2017)**	UK	Chronic Fatigue Syndrome	To further understanding of the factors predicting patient activity patterns in CFS. patterns.	Prospective, ESM	Clinical	N = 23;<br>Mean age (SD) = 35.5 (13.96);<br>% Female = NR	Research	aRMT only. ClinTouch software	6 days		X
**Bandarian-Balooch et al. (2017)**	Australia	Chronic headache & migraine	**To test the reliability and validity of a newly developed e-diary for headaches, and to compare this to 7-item paper diary.**	Partially randomised trial	Community	N = 181;<br>Mean age (SD) = 26.52 (10.15);<br>% Female = 80.6	Research	aRMT only. E-diary app developed by authors	30 days		X

**Barnett et al. (2018)**	US	Schizophrenia	To explore the potential of active and passive smartphone data to predict relapse in schizophrenia.	Prospective, observational	Clinical	N = 17;<br>Mean age (SD) = NR (NR);<br>% Female = NR	Research	Combined. The Beiwe app, as part of the Beiwe research platform	3 months		X
**Bauer et al. (2018a)**	US	Anxiety/Depression	**A pilot study to assess the feasibility, acceptability and fit of the mobile health platform with the Collaborative Care workflow**.	Pilot, prospective, observational, mixed-methods	Clinical	N = 17;<br>Mean age (SD) = NR (NR);<br>% Female = 59	Clinical Support	Combined. Ginger.io app, paired with a web-based provider dashboard	12 weeks		X
**Bauer et al. (2018b)**	US	Depression	To examine the association between the PHQ-9 and a daily depressive symptom measure, and to compare how these perform in predicting patient outcomes.	Secondary analysis of an existing dataset	Community	N = 547;<br>Mean age (SD) = 33 (11);<br>% Female = 78.3	Research	Combined. Ginger.io app, ACE cognitive assessment app	8 weeks		X
**Beiwinkel et al. (2016)**	Germany	Bipolar	To investigate whether smartphone measurements predicted clinical symptoms levels and clinical symptom change.	Pilot, prospective, observational	Clinical	N = 13;<br>Mean age (SD) = 47.2 (3.8);<br>% Female = 38.5	Research	Combined. SIMBA app	12 months		X
**Ben-Zeev et al. (2014)**	US	Schizophrenia	**To test the first deployment of a smartphone intervention (FOCUS system) for schizophrenia.**	Field trial	Clinical	N = 33;<br>Mean age (SD) = 45.9 (8.78);<br>% Female = 39	Self-management	aRMT only. The FOCUS smartphone system	1 month		X
**Block et al. (2019)**	Switzerland	Insomnia in Major Depressive Disorder and Social Phobia	To explore whether anticipatory stress and state openness and engagement affected subsequent sleep quality over the course of a week.	Quasi-experimental longitudinal design with an ESM paradigm	Clinical and community	N = 290;<br>Mean age (SD) = 31.8 (11.5);<br>% Female = 66.5	Research	aRMT only. Author-developed app	7 days		X

**Bove et al. (2015)**	US	Multiple Sclerosis	**To investigate the feasibility of performing frequent smartphone-based assessments of patients with MS and their cohabitants.**	Case-control with matched pairs	Clinical	N = 76;<br>Mean age (SD) = 25.1 (10.2) ;<br>% Female = 74	Research	Combined. Custom application suite	1 year	X	X
**Broderick et al. (2019)**	US	Cancer	**To examine the correspondence of PROs with actigraphy to determine the criterion validity of PROs to reflect activity level.**	Pilot feasibility study, single-arm observational	Clinical	N = 65;<br>Mean age males (SD) = 43.6 (11.6);<br>Mean age females (SD) = 52.8 (11.7)<br>% Female = 50	Research	Combined. Author-developed app Microsoft Band 2	60 days		X
**Buck et al. (2019)**	US	Persecutory ideation in Schizophrenia	To (1) quantify between- and within-person variability in PI, (2) evaluate pre-existing models of indicators of PI with EMA gathered over a longer timespan than extant studies, and (3) identify passively sensed indicators of PI.	Observational	Clinical	N = 62;<br>Mean age (SD) = NR (NR);<br>% Female = NR	Research	Combined. CrossCheck mobile health assessment system	1 year		X
**Busk et al. (2020a)**	Denmark	Bipolar Disorder	**To examine the feasibility of producing daily estimates of clinical ratings of depression and mania based on smartphone self-assessments of symptoms.**	Secondary data analysis from MONARCA II RCT dataset	Clinical	N = 84;<br>Mean age (SD) = 43.1 (12.4);<br>% Female = 61.9	Research	aRMT only. Monsenso system app	9 months		X
**Busk et al. (2020b)**	Denmark	Bipolar Disorder	**To examine the feasibility and technical foundation of forecasting daily mood scores in bipolar disorder based on daily smartphone self-assessments.**	Secondary data analysis from MONARCA II RCT dataset	Clinical	N = 84;<br>Mean age (SD) = 43.1 (12.4);<br>% Female = 62	Research	aRMT only. Monsenso system app	9 months		X

**Carpenter et al. (2019)**	US	Chronic Lower Back Pain	To examine the bidirectional relationship of prescribed opioid use and aversive states over time in the lives of chronic pain patients.	Observational EMA	Clinical and community	N = 34;<br>Mean age (SD) = 45.3 (8.8);<br>% Female = 76.5	Research	aRMT only.<br>Unnamed EMA app	14 days		X
**Carr et al. (2018a)**	UK	Bipolar Disorder, Borderline Personality Disorder	To examine whether the disruption of the synchrony of diurnal physiology would be disturbed in BD in comparison to BPD.	Observational, 3 groups (BD, BPD, healthy controls)	Community	N =129;<br>Mean age BD (SD) = 39 (12.9);<br>Mean age BPD (SD) = 33.6 (10.4);<br>Mean age HC (SD) = 36.6 (13.0);<br>% Female = 74	Research	Combined.<br>MoodZoom app<br>Proteus patch	1 week		X
**Carr et al. (2018b)**	UK	Bipolar Disorder, Borderline Personality Disorder	To (a) develop a method of quantifying diurnal rhythm regularity, (b) explore how diurnal variability of activity, sleep, HR and variability in mood are altered in BD, BPD and HC (c) investigate the links between diurnal variability and mood variability in BD and BPD participants	Observational, 3 groups (BD, BPD, healthy controls)	Community	N =141;<br>Mean age BD (SD) = 41 (11.4);<br>Mean age BPD (SD) = 34.3 (10.2);<br>Mean age HC (SD) = 43.4 (14.6);<br>% Female = 78	Research	Combined.<br>MoodZoom app<br>Proteus patch	1 week		X
**Chan et al. (2019)**	HK	Irritable Bowel Syndrome	To model the interaction between positive emotions, negative emotions, daily life stress, bowel symptoms in IBS	ESM	Clinical	N = 57;<br>Mean age IBS (SD) = 37.7 (12.8);<br>Mean age HC (SD) = 39.2 (12.9)<br>% Female = 60	Research	aRMT only.<br>ESM app adapted for IBS patients	14 days		X
**Corden et al. (2016)**	US	Depression	**To evaluate the feasibility of a systemic digital intervention, MedLink.**	Pilot feasibility, field trial	Clinical	N = 11;<br>Mean age (SD) = 45.1 (16.7);<br>% Female = 73	Clinical Support	Combined.<br>MedLink app<br>Wisebox pill box	8 weeks	X	X

**Croutham el et al. (2018)**	US/UK	Rheumatoi d Arthritis	**To assess the feasibility of using an entirely digital approach to conduct a real-world observational study of patients with rheumatoid arthritis.**	Prospective, real-world observational	Community	N = 399; Mean age (SD) = 47.9 (12.3); % Female = 80.7	Research	Combined. PARADE app, as part of ResearchKit infrastructure	12 weeks	X	X
**Faurholt-Jepsen et al. (2014)**	Denmark	Bipolar Disorder	To investigate possible correlations between clinically rated depressive and manic symptoms of bipolar disorder and subjective and objective Smartphone data, respectively	Prospective, observational, pilot	Clinical	N = 17; Mean age (SD) = 33.4 (9.5); % Female = 70.6	Research	Combined. MONARCA smartphone software	3 months		X
**Faurholt-Jepsen et al. (2015a)**	Denmark	Bipolar Disorder	To investigate differences in proportion of time with different measures of illness activity between patients with bipolar type I and bipolar disorder type II.	Secondary analyses on MONARCA RCT dataset	Clinical	N = 33; Mean age (SD) = 29.1 (7.4); % Female = 71	Research	Combined. MONARCA smartphone software	310 days		X
**Faurholt-Jepsen et al. (2015b)**	Denmark	Bipolar Disorder	To evaluate the hypothesis that daily electronic self-monitoring using the MONARCA system developed for Android smartphones, including a clinical feedback loop, in patients with bipolar disorder would reduce depressive and manic symptoms.	Randomized, placebo-controlled, single-blinded, parallel group design	Clinical	N = 78; Mean age intervention (SD) = 29.1 (7.5); Mean age control (SD) = 29.5 (9.4); % Female = 67.2	Research	aRMT only. MONARCA smartphone software	6 months		X
**Faurholt-Jepsen et al. (2016a)**	Denmark	Bipolar Disorder	To examine whether voice features extracted during phone calls from everyday life in naturalistic settings	Naturalistic, observational	Clinical	N = 28; Mean age (SD) = 30.3 (9.3); % Female = 65.4	Research	Combined. MONARCA smartphone software (with added	12 weeks		X

			can discriminate between affective states.					voice recording)			
**Faurholt-Jepsen et al. (2016b)**	Denmark	Bipolar Disorder	To examine whether the level of detailed automatically generated objective smartphone data collected from smartphones correlates with the level of depressive and manic symptoms assessed.	Prospective, observational (MONARCA study)	Clinical	N = 29;<br>Mean age (SD) = 30.2 (8.8);<br>% Female = 62	Research	Combined. MONARCA smartphone software	12 weeks		X
**Faurholt-Jepsen et al. (2019a)**	Denmark	Bipolar Disorder	**To examine the feasibility and usability of two different smartphone-based monitoring systems.**	RCT across 3 sites	Clinical	N = 60;<br>Age range = 18-69;<br>% Female = 68.3	Self-management	Combined. PULSO system with Fitbit accelerometer TRILOGIS-MONSENSO with Nokia health accelerometer	4 weeks		X
**Faurholt-Jepsen et al. (2019b)**	Denmark	Bipolar Disorder	To use daily smartphone-based self-monitoring data to investigate differences in mood, euthymia and mood instability between patients with bipolar disorder type I and type II.	Prospective, observational	Clinical	N = 84;<br>Mean age (SD) = 43.0 (12.2);<br>% Female = 61.2	Research	Combined. Monsenso system iPhone	9 months		X
**Faurholt-Jepsen et al. (2019c)**	Denmark	Bipolar Disorder	To investigate the association between mood instability and perceived stress, quality of life, and functioning in patients with bipolar disorder.	Prospective, observational	Clinical	N = 84;<br>Mean age (SD) = 43.0 (12.2);<br>% Female = 61.2	Research	Combined. Monsenso system iPhone	9 months		X

**Faurholt-Jepsen et al. (2020)**	Denmark	Bipolar Disorder	To investigate whether smartphone-based monitoring and mood prediction including a clinical feedback loop in patients with BD would reduce the level of depressive and manic symptoms more than standard treatment.	Randomized controlled, single-blind, parallel-group trial	Clinical	N = 129;<br>Mean age intervention (SD) = 43.0 (12.4);<br>Mean age control (SD) = 43.2 (12.4);<br>% Female = 57.9	Self-management	Combined.<br>Monsenso system<br>iPhone	9 months		X
**Garcia-Palacios et al. (2014)**	Spain	Fibromyalg ia	**To compare the accuracy and acceptability of an RTDC assessment method running on a smartphone in a single-centre, randomized, crossover study with fibromyalgia sufferers.**	Single-centre, randomized, crossover	Clinical	N = 47;<br>Mean age (SD) = 48.05 (8.0);<br>% Female = 100	Research	aRMT only.<br>Author-developed F-EMA software application	7 days x 2	X	X
**Greer et al. (2020)**	US	Cancer	To test the use of a smartphone mobile app to promote adherence to oral therapy for cancer and symptom management.	1:1 parallel-assignment RCT	Clinical	N = 181;<br>Mean age (SD) = 53.3 (12.9);<br>% Female = 53.6	Clinical Support	Combined.<br>Author-developed app<br>Fitbit integration<br>Electronic pill caps	12 weeks	X	X
**Gustavell et al. (2019)**	Sweden	Cancer (Pancreatic and Periampulla ry)	The evaluate the impact on health-related quality of life and self-care activity when using the Interaktor app following pancreaticoduodene ctomy due to cancer.	Historically-controlled, single center	Clinical	N = 59;<br>Mean age intervention (SD) = 67 (8.7);<br>Mean age control (SD) = 66 (8.8);<br>% Female = 37	Self-management	aRMT only.<br>Interaktor app	6 months		X
**Gustavell et al. (2020)**	Sweden	Pancreatic Cancer	**To describe how patients used the Interaktor app following pancreaticoduoden ectomy due to cancer and their experience with doing so.**	Prospective, observational	Clinical	N = 26;<br>Mean age (SD) = 67 (878);<br>% Female = 31	Clinical Support	aRMT only.<br>Interaktor app	4 weeks+	X	X

**Hidalgo-Mazzei et al. (2016)**	Spain	Bipolar Disorder	**To evaluate, during 3 months, acceptability, safety and satisfaction of the SIMPLe smartphone application designed to monitor symptoms in BD.**	Feasibility	Clinical	N = 51;<br>Mean age (SD) = 43.9 (11.4);<br>% Female = 42.9	Self-management	aRMT only. SIMPLe 1.0 smartphone app	3 months	X	X
**Hung et al. (2016)**	China	Depression	**To examine the validity of smartphone-based EMA for depression in Chinese patients and explored the determinants of use.**	Prospective, observational	Clinical	N = 59;<br>Mean age (SD) = 37.9 (13.9)<br>% Female = 63	Research	aRMT only. iHOPE smartphone app	8 weeks		X
**Ireland & Andrews (2019)**	Australia	Chronic pain	To provide a summary of the portal and mobile app ROADMAP, and results from a recent pilot study.	Pilot	Clinical	N = 20;<br>Mean age range = 20-70;<br>% Female = NR	Clinical Support	Combined. Pain ROADMAP mobile health platform (app + wearable device)	3 x 1 week		X
**Jamison et al. (2017)**	US	Chronic Pain	**To determine the effect of introducing a smartphone pain app to chronic pain patients that assesses, monitors, and communicates their status to their providers, and provides self-management strategies.**	Pilot RCT	Clinical	N = 90;<br>Mean age (SD) = 47.1 (13.5);<br>% Female = 63.8	Clinical Support	Combined. Author-developed smartphone pain app Fitbit	3 months	X	X
**Jamison et al. (2018a)**	US	Chronic Pain	**To determine the feasibility, tolerability, safety and efficacy of a smartphone pain app among chronic pain patients over a six-month trial.**	Longitudinal trial	Clinical	N = 90;<br>Mean age (SD) = 46.7 (12.9);<br>% Female = 64.4	Self-management	Combined. Author-developed smartphone pain app Fitbit	6 months	X	X

**Jamison et al. (2018b)**	US	Osteoarthritis	To investigate the efficacy of vibration technology for women with hand pain due to osteoarthritis.	RCT	Clincal	N = 69; Mean age (SD) = 63 (7.8); % Female = 100	Research	aRMT only. Author-developed smartphone pain app Vibrating glove	3 months		X
**Juengst et al. (2019)**	USA	Traumatic Brain Injury	To investigate within-person variability in daily self-reported emotional and fatigue symptoms and factors associated with high within-person variability among individuals with chronic traumatic brain injury (TBI).	Prospective, descriptive, pilot study, secondary analysis	Community	N = 18; Mean age (SD) = 38.3 (12.7); % Female = 44	Research	aRMT. Smartphone app (not described)	8 weeks		
**Kauer et al. (2012) LINK WITH REID**	Australia	Adolescent Depression	To investigate the effect of monitoring mood, stress, and coping strategies on Emotional Self-Awareness and depressive symptoms.	Single-blind RCT	Clinical	N = 118; Mean age intervention (SD) = 18.5 (3.2); Mean age control (SD) = 17.4 (3.2); % Female = 70.5	Clinical Support	aRMT. Mobiletype program version 4	2-4 weeks		X
**Kearney et al. (2009)**	UK	Cancer (Breast, Lung, Colorectal)	To evaluate the impact of a mobile phone-based, remote monitoring, advanced symptom management system (ASyMS©) on the incidence, severity and distress of six chemotherapy-related symptoms.	RCT	Clinical	N = 112; Mean age (SD) = 56.0 (10.5); % Female = 76.8	Clinical Support	aRMT only. ASyMS© mobile-based system	4 cycles of chemotherapy		X
**Kim et al. (2016)**	South Korea	Depression in Breast Cancer	**To evaluate the potential of a mobile mental-health tracker that uses three daily mental-health ratings as indicators for depression.**	Observational, prospective	Clinical	N = 85; Mean age (SD) = 44.4 (7.0); % Female = 100	Clinical Support	aRMT only. Pit-a-Pat mobile app	48 weeks	X	X

**Lenaert et al. (2020)**	Netherlands	Stroke	To investigate whether post-stroke fatigue was predicted by type of activity and physical activity measured at the same moment and at earlier time points.	Longitudinal, observational	Clinical	N =30; Mean age (SD) = 55.3 (7.6); % Female = 50	Research	aRMT only. PsyMat smartphone app	6 days		X
**Li et al. (2019)**	USA	Bipolar Disorder	To study within-person variability in mood, cognition, energy, and impulsivity measured in an Ecological Momentary Assessment paradigm in bipolar disorder by using modern statistical techniques.	Secondary analysis of prior study (observational, prospective, two-arm, parallel group)	Community	N = 20; Mean age (SD) = NR (NR); % Female = NR	Research	aRMT only. Mode of delivery NR	14 days		
**Lin et al. (2019)**	USA	Chronic Lower Back Pain	**To assess the feasibility and usability of an ecological momentary assessment smartphone application collecting real-time data on chronic low back pain.**	Pilot, prospective	Clinical	N = 18; Mean age (SD) = 50.7 (17.7); % Female = 67	Research	aRMT only. Earpress smartphone app	4 weeks	X	X
**Moore et al. (2017)**	USA	Older Adults with HIV	**To test the feasibility and acceptability of using EMA methods delivered via smartphones to assess daily functioning and other behavioral factors among older HIV+ adults.**	Prospective, observational	Community	N = 20; Mean age (SD) = 58.8 (4.3); % Female = 15	Research	aRMT only. Android smartphone delivered surveys	1 week	X	X

**Moukaddam et al. (2019)**	US	Depression and Anxiety	To formally examine the correlation between objective clinician-administered measures for depression and anxiety and data collection based on passive sensors.	Pilot, prospective	Clinical	N =25; Mean age (SD) = 50.3 (10.1); % Female = 76	Self-management	Combined. SOLVD smartphone app	8 weeks		X
**Niendam et al. (2018)**	USA	Adolescents with Early Psychosis	To determine the validity, feasibility and acceptability of implementing a smartphone application in adolescents and young people with early psychosis.	Longitudinal, within-person	Clinical	N = 76; Mean age (SD) = 18.8 (3.7); % Female = 34	Clinical Support	Combined. Ginger.io smartphone app	Up to 14 months	X	X
**Palmius et al. (2018)**	UK	Depression	To demonstrate the utility of splitting the population into subsets of individuals that exhibit similar relationships between their objective markers and their mental states.	Observational	Community	N = 59; Mean age (SD) = 41 (15.8); % Female = 75	Self-management	Combined. Author-developed Android-based app Wearable devices	6 weeks+		X
**Paolillo et al. (2018)**	US	HIV	To examine real-time relationships between social activity and mood, fatigue, and pain in a sample of older people living with HIV.	Prospective, observational, EMA	Community	N = 20; Mean age (SD) = 58.8 (4.3); % Female = 15	Research	aRMT only. Android smartphone	1 week		X
**Perez Arribas et al. (2018)**	**UK**	Bipolar Disorder and Borderline Personality Disorder	To accurately classify participants within their diagnostic categories and predict mood using daily reports.	Prospective, observational, case-control	Community	N = 139; Mean age BD (SD) = 38 (21); Mean age BPD (SD) = 34 (15); Mean age HC (SD = 37 (20); % Female = 72.3	Research	aRMT only. MoodZoom smartphone app	3 months- 1 year		X
**Prada et al. (2017)**	Switzerland	Borderline Personality Disorder	**To assess the usability and acceptability of a**	Longitudinal, pilot	Clinical	N = 16; Mean age (SD) = 30.6 (9.3);	Self-management	aRMT only.	6 months	X	X

			**smartphone app for ambulatory monitoring and reduction of aversive tension in BPD subjects.**			% Female = 100		EMOTEO smartphone app			
**Probst et al. (2017)**	Germany	Tinnitus	To explore whether tinnitus loudness and tinnitus distress fluctuate within a 24-h interval.	Prospective	Community	N = 350;<br>Mean age (SD) = 45.4 (12.1);<br>% Female = 26.9	Self-management	aRMT only. Track YourTinnitus smartphone app	NR		X
**Reid et al. (2011)**	Australia	Depression, Anxiety, Stress	To investigate the benefits and utility of the *mobiletype* program.	Single-blind RCT -	Clinical	N = 118;<br>Mean age intervention (SD) = 18.5 3.2);<br>Mean age control (SD) = 17.4 (3.2);<br>% Female = 80.4	Clinical Support	aRMT only. Mobiletype program version 4	2-4 weeks		X
**Reid et al. (2013)**	Australia	Depression, Anxiety, Stress	To examine the *mobiletype* program as i) a clinical assistance tool, ii) doctor-patient rapport and, iii) pathways to care.	Secondary outcomes of a larger RCT	Clinical	N = 118;<br>Mean age (SD) = 18.1 (3.2);<br>% Female = 71.9	Clinical Support	aRMT only. Mobiletype program version 4	2-4 weeks		X
**Rijsbergen et al. (2020)**	Netherlands	Skin Diseases	**To investigate the utility of an electronic diary application for patients with skin diseases in outpatient clinical trials.**	6 randomised, double-blind, placebo controlled clinical trials	Clinical	N = 256;<br>Mean age (SD):<br>Trial 1 = 25.8 (10.6)<br>Trial 2 = 24.9 (7.8)<br>Trial 3 = 24.4 (6.5)<br>Trial 4 = 34.4 (11.6)<br>Trial 5 = 30.8 (10.6)<br>Trial 6 = 49.8 (11.0);<br>% Female = 58.3	Self-management	aRMT only. e-diary smartphone app	4-12 weeks	X	X
**Rodriguez Hermosa et al. (2020)**	Spain	Chronic Obstructive Pulmonary Disease (COPD)	**To provide further evidence in support of prospectively recording daily symptoms as a useful strategy to detect COPD exacerbations through the smartphone app, Prevexair, and to analyze daily compliance.**	Multicentre, prospective, cohort	Clinical	N = 126;<br>Mean age (SD) = 66.1 (8.1);<br>% Female = 21.6	Self-management	aRMT only. Prevexair smartphone app	2 weeks	X	X

**Ross et al. (2020)**	USA	Chronic Pain	**To determine the long-term effects of using a smartphone pain app that offers pain management strategies and allows patients with chronic pain to assess, monitor, and communicate their condition to their health care providers.**	Secondary analysis of a dataset	Clinical	N = 253; Mean age (SD) = 51.5 (13.7); % Female = 72.8	Research	aRMT only. Author-developed pain app	3 months	X	X
**Schwartz et al. (2016)**	USA	Bipolar Disorder	**To examine the feasibility of symptom tracking in bipolar disorder.**	Pilot, two-arm, parallel group, observational	Community	N = 20; Mean age BD (SD) = 48.9 (16.8); Mean age HC (SD) = 39.7 (14.2) % Female = 75	Research	aRMT only. Motorola smartphone	14 days	X	X
**Selter et al. (2018)**	USA	Chronic Lower Back Pain	**To (1) describe patient engagement with the Limbr program, (2) describe patient-perceived utility, and (3) assess its validity for quantifying functional status among patients with chronic lower back pain.**	Single-arm trial	Clinical	N = 35; Mean age (SD) = 46 (16); % Female = 63	Self-management	Combined. Smartphone app suite of 5 apps	3 months	X	X
**Seng et al. (2018)**	USA	Headache	**To describe user adherence to a smartphone-based headache diary, and to evaluate the relationships between level of anxiety and adherence to the electronic headache diary between individuals.**	Naturalistic, longitudinal, cohort	Clinical and community	N = 1561; Mean age (SD) = 39 (13.2); % Female = 88.1	Self-management	aRMT only. Curelator Headache app	90 days	X	X

**Sengupta et al. (2020)**	USA	Coronary Heart Disease	**To examine the usability of a prototypic mHealth intervention (HerBeat) for women with CHD.**	Single-group, pretest, posttest design	Clinical	N = 10;<br>Mean age (SD) = 64.4 (6.3);<br>% Female = 100	Self-management	Combined. Smartphone app<br>Moto 360 smartwatch	12 weeks		X
**Shafran et al. (2019)**	UK	Anxiety and Depression	To evaluate whether monitoring symptoms of anxiety and/or depression increased reported actual receipt of treatment.	RCT	Community	N = 306;<br>Mean age (SD) = 27.2 (9.7);<br>% Female = 70.9	Research	aRMT only. Mood Mate smartphone app	30 days		X
**Stanislaus et al. (2020)**	Denmark	Bipolar Disorder	To investigate differences in smartphone-based self-reported mood instability between BD patients, unaffected relatives and healthy controls.	Longitudinal, observational	Clinical (vs healthy controls)	N = 203;<br>Mean age BD (SD) = 28 (NR);<br>Mean age UR (SD) = 26 (NR);<br>Mean age HC (SD) = 26 (NR);<br>% Female = 63	Self-management	aRMT only. Monsenso smartphone app and web-based interface	Up to 800 days		X
**Suso-Ribera et al. (2018)**	Spain	Pain	**To explore the validity, reliability, feasibility, and usefulness of Pain Monitor in adults with chronic pain.**	Repeated-measures, observational	Clinical	N = 38;<br>Mean age (SD) = 42.7 (9.9);<br>% Female = 52.6	Research	aRMT only. Pain Monitor smartphone app	30 days	X	X
**Taft et al. (2018)**	Sweden	Hypertension	To explore relationships between patients' self-monitoring of blood pressure and their concurrent self-reports of medication intake, well-being, stress, physical activity and symptoms.	Secondary analysis of a prospective study	Clinical	N = 50;<br>Mean age (SD) = 59.5 (NR);<br>% Female = 48	Self-management	aRMT only. Mobile-based self-management support system	8 weeks		X
**Torous et al. (2015)**	USA	MDD	**To investigate adherence among psychiatric outpatients diagnosed with major depressive disorder in utilizing their personal smartphones to run a custom app to**	Observational, prospective	Clinical	N = 13;<br>Mean age males (SD) = 48 (16);<br>Mean age females (SD) = 35 (13);<br>% Female = 77	Research	aRMT only. Mindful Moods smartphone app	30 days	X	X

			**monitor depression symptoms.**								
**Tsanas et al. (2016)**	UK	Bipolar and Borderline Personality Disorder	To introduce and validate a novel clinical questionnaire used for daily mood monitoring as part of a smartphone application.	Longitudinal, case- control	Community	N = 130;<br>Mean age BD (SD) = 38 (21);<br>Mean age BPD (SD) = 34 (15);<br>Mean age HC (SD) = 37 (20);<br>% Female = 72	Research	aRMT only. Mood Zoom smartphone app	3-12 months		X
**Van Til et al. (2020)**	USA	Bipolar Disorder	**To compare adherence to active and passive monitoring in participants using a smartphone app to log symptoms and an activity tracker to track sleep, activity, and heart rate.**	Prospective	Community	N = 47;<br>Mean age (SD) = 41.9 (10.8);<br>% Female = 53.1	Research	Combined. Smartphone app<br>Fitbit Alta HR	6 weeks	X	X
**Vork et al. (2020)**	Netherlands	Irritable Bowel Syndrome	To evaluate the association between stress and abdominal pain, using the Experience Sampling Method (ESM) as a real-time, repeated measurement method.	Prospective, observational	Clinical	N = 73;<br>Mean age IBS (SD) = 36.7 (NR);<br>Mean age HC (SD) = 31.1 (NR);<br>% Female = 68.5	Self-management	aRMT only. MEASuRE smartphone app	7 days		X
**Wu & Cronin-Golomb (2020)**	US	Parkinson's Disease	To assess sleep as a possible treatment target for improving daytime functioning in PD.	Prospective, feasibility	Community	N = 20;<br>Mean age (SD) = 66.5 (9.3);<br>% Female = 35	Self-management	Combined. Sym Trend smartphone app	14-15 days		X
**Yang et al. (2019)**	South Korea	Moyamoya Disease	**To examine factors affecting completion rates of EMA using a mobile application for adult patients**	Prospective, observational	Clinical	N = 98;<br>Mean age (SD) = 41 (10.3);<br>% Female = 69.4	Research	aRMT only. Smartphone app	7 days	X	X

			**with Moyamoya disease.**								
**Zia et al. (2016)**	USA	Irritable Bowel Syndrome	**To evaluate the feasibility and usability of a novel food and symptom journal app, specifically designed for patients with IBS.**	Prospective, pilot	Clinical and community	N = 11;<br>Mean age (SD) = 35 (11);<br>% Female = 73	Self-management	aRMT only. Author-developed IBS smartphone app	2 weeks	X	X

NR= not reported. HC= healthy controls. Studies with bold aim noted engagement as a main aim.
